# Quantitative Trait Locus Mapping of Flowering Time and Maturity in Soybean Using Next-Generation Sequencing-Based Analysis

**DOI:** 10.3389/fpls.2018.00995

**Published:** 2018-07-11

**Authors:** Lingping Kong, Sijia Lu, Yanping Wang, Chao Fang, Feifei Wang, Haiyang Nan, Tong Su, Shichen Li, Fengge Zhang, Xiaoming Li, Xiaohui Zhao, Xiaohui Yuan, Baohui Liu, Fanjiang Kong

**Affiliations:** ^1^School of Life Sciences, Guangzhou University, Guangzhou, China; ^2^The Key Laboratory of Soybean Molecular Design Breeding, Northeast Institute of Geography and Agroecology, Chinese Academy of Sciences, Harbin, China; ^3^Mudanjiang Branch of Heilongjiang Academy of Agricultural Sciences, Mudanjiang, China; ^4^University of Chinese Academy of Sciences, Beijing, China

**Keywords:** soybean, flowering time, maturity, reproduction period, quantitative trait loci

## Abstract

Soybean (*Glycine max* L.) is a major legume crop that is mainly distributed in temperate regions. The adaptability of soybean to grow at relatively high latitudes is attributed to natural variations in major genes and quantitative trait loci (QTLs) that control flowering time and maturity. Identification of new QTLs and map-based cloning of candidate genes are the fundamental approaches in elucidating the mechanism underlying soybean flowering and adaptation. To identify novel QTLs/genes, we developed two F8:10 recombinant inbred lines (RILs) and evaluated the traits of time to flowering (R1), maturity (R8), and reproductive period (RP) in the field. To rapidly and efficiently identify QTLs that control these traits, next-generation sequencing (NGS)-based QTL analysis was performed. This study demonstrates that only one major QTL on chromosome 4 simultaneously controls R1, R8, and RP traits in the Dongnong 50 × Williams 82 (DW) RIL population. Furthermore, three QTLs were mapped to chromosomes 6, 11, and 16 in the Suinong 14 × Enrei (SE) RIL population. Two major pleiotropic QTLs on chromosomes 4 and 6 were shown to affect flowering time, maturity, and RP. A QTL influencing RP was identified on chromosome 11, and QTL on chromosome 16 was associated with time to flowering responses. All these QTLs contributed to soybean maturation. The QTLs identified in this study may be utilized in fine mapping and map-based cloning of candidate genes to elucidate the mechanisms underlying flowering and soybean adaptation to different latitudes and to breed novel soybean cultivars with optimal yield-related traits.

## Introduction

Flowering time and maturity traits play crucial roles in economic crop production. An intricate network with various (epi-)genetic regulators responding to environmental and endogenous triggers controls the timely onset of flowering and maturity in plants. The pleiotropic effects on important agronomic characters influence adaptation to new geographical/climatic conditions and future perspectives for crop improvement (Blümel et al., 2015). Soybean (*Glycine max* L.) is a major legume crop that is mainly distributed in temperate regions, and days to flowering and maturity are key factors for developing soybean cultivars with a wider geographical adaptation (Lu et al., 2017). Flowering time and reproductive period (RP) greatly impact soybean maturity; however, RP is also an important soybean trait that is closely related to yield, seed quality, and tolerance to various environmental stresses (Xu et al., 2013). Both time of flowering and maturity in soybean are quantitative traits that are controlled by multiple genes. To date, 12 major genes/loci related to time of flowering and maturity [*E1* and *E2* (Bernard, 1971), *E3* (Buzzell, 1971), *E4* (Buzzell and Voldeng, 1980; Saindon et al., 1989a,b), *E5* (McBlain and Bernard, 1987), *E6* (Bonato and Vello, 1999), *E7* (Cober and Voldeng, 2001), *E8* (Cober et al., 2010), *E9* (Kong et al., 2014; Zhao et al., 2016), *E10* (Samanfar et al., 2017), *J* (Ray et al., 1995), and *Dt1* (Liu et al., 2010; Tian et al., 2010)] have been reported in soybean. A previous study has shown that no unique *E5* gene exists and has been misidentified by unexpected outcrossing contamination of the *E2* locus (Dissanayaka et al., 2016). Test crossing, genetic mapping, and sequencing suggest that the *E6* and *J* loci might be tightly linked (Li et al., 2017). *E7*, which is situated on MLG C2, has been reported in linkage with *E1* (Molnar et al., 2003). Genes within the maturity loci *E1–E4*, *E9*, *E10*, *J*, and *Dt1* have been identified by map-based cloning, and their functions have been characterized (Liu et al., 2008, 2010; Watanabe et al., 2009, 2011; Tian et al., 2010; Xia et al., 2012; Zhao et al., 2016; Samanfar et al., 2017; Lu et al., 2017); however, candidate genes within the maturity-related *E6, E7*, and *E8* loci remain unknown.

Although the functions of some flowering loci/genes have been characterized, current understanding of the underlying mechanism of time to flowering and maturity in soybean remains limited. The *E1* family genes have only been reported in legumes, and the failure to detect a specific phenotype that is caused by the overexpression of *E1* in Arabidopsis and rice suggests that the exogenous *E1* gene is independent of the regulatory networks of photoperiodic flowering in these species (Zhang et al., 2016). The flowering regulatory pathway in soybean may be distinct from that in model plants such as Arabidopsis and rice (Zhang et al., 2016). *E1* is a key gene in the regulatory network of flowering in soybean. It is negatively correlated to *GmFT2a* and *GmFT5a*, which are homologs of FLOWERING LOCUS T that promotes flowering (Xia et al., 2012). *J* the ortholog *of A. thaliana* EARLY FLOWERING 3 (*AtELF3*). The J protein physically associates with the *E1* promoter to downregulate its transcription, relieving repression of two important *FT* genes and promoting flowering under short-day conditions. In addition, *J* might also function at least partially downstream of *E3* and *E4* under short-day conditions (Lu et al., 2017). The function of *J* under long-day conditions remains unclear. The exact molecular function of flowering- and maturity-related genes remain elusive. Therefore, identification of novel loci or genes for photoperiodic flowering and maturity may improve our understanding of soybean flowering and adaptation to different latitudes. Different soybean genetic resources and populations have been used to identify novel QTLs of important agronomic traits using multiple approaches. One of these, next-generation sequencing (NGS), is a powerful method for the identification of single-nucleotide polymorphism (SNP) markers on a large scale for the construction of a high-density genetic map for QTL mapping (Zhou et al., 2016). In this study, we used diverse genetic resources to develop two RIL populations as well as the NGS-based approach to identify QTLs for photoperiodic flowering and maturity.

## Materials and Methods

### Plant Materials and Field Trials

The two F_8:10_ RILs used in mapping were developed using the single-seed descent method. One RIL population that consisted of 140 genotypes was developed from a cross between cultivars Suinong 14 (*E1e2e3E4Dt1*) and Enrei (*E1e2e3E4dt1*) and designated as SE. The other RIL population consisted of 126 genotypes and was developed from a cross between cultivars Dongnong 50 (*e1^as^E2E3E4Dt1*) and Williams 82 (*e1^as^E2E3E4Dt1*) and designated as DW.

The F_8:9_ seeds of both RILs that were used for mapping were grown in the experimental field in Harbin (45°43′N, 126°45′E), China in May 2016 and Mudanjiang (44°36′N, 129°35′E), China in May 2016. The F_9:10_ seeds were grown in Harbin, China in May 2017. The seeds of each RIL genotype and the parental lines were planted at a row length of 5 m, row space of 60 cm, and plant distance from each other of about 20 cm. There were about 25 plants in each row. Standard cultivation practices to control insects and weeds were used for all trials.

The date of emergence of the -25 plants in each row was recorded. Each plant was assigned a number for identification purposes. The flowering and maturity time of each plant was recorded, and the length of the RP was calculated. Days to flowering were recorded at the R1 (Fehr et al., 1971) stage (days from emergence to first open flower in 50% of the plants). Days to maturity were recorded at the R8 (Fehr et al., 1971) stage (95% of the pods have turned their mature color in 50% of the plants). Day length of reproduction period (RP) were recorded as R8 minus R1 (RP = R8 – R1). One-way ANOVA was used to test the significance of the differences in all traits between parents. SPSS18.0 (SPSS Inc., Chicago, IL, United States) was also used for correlation analysis, descriptive statistics, and two-way ANOVA analysis using R1, R8, and RP trait data of RILs in different environments. Broad-sense heritability (*h*^2^_b_) was estimated for three traits in combined environments (R1 of year 2016 in Mudanjiang and Harbin and 2017 in Harbin; R8 and RP of year 2016 and 2017 in Harbin) according to the following equation: *h*^2^_b_ = VG/(VG + VE), where VG and VE are estimated using QTLNetwork 2.1 (Yang et al., 2008).

### DNA Extraction

Young and fully developed trifoliate leaves from parents and the RIL individuals were collected and frozen in liquid nitrogen and then transferred to a -80°C freezer. Total genomic DNA was extracted from each parental and RIL leaf sample using the CTAB DNA extraction method. The integrity and quality of the extracted DNA were evaluated by 1% agarose gel electrophoresis. DNA concentrations of each sample were determined using a Qubit^®^ 2.0 Fluorometer (Invitrogen, Carlsbad, CA, United States) and NanoDrop 2000 (Thermo Scientific, Wilmington, DE, United States).

### Genotyping by High-Throughput Sequencing

For each of the four parents, a total of 1.5 μg of the DNA sample were prepared for whole genome resequencing. Sequencing libraries were generated as described by Cheng et al. (2015). These parental libraries were sequenced on an Illumina HiSeq 2000 platform (Illumina, Inc., San Diego, CA, United States), and 125-bp paired-end reads with insert sizes of around 350 bp were generated.

The SE population was genotyped using specific-locus amplified fragment sequencing (SLAF), and the DW population was genotyped using the genotyping-by-sequencing (GBS) technology. Based on the reference parental polymorphic loci, genotypes of SNPs were identified by low-coverage sequencing of the two RIL populations (Huang et al., 2009; Davey et al., 2013).

For the DW RIL population, genomic DNA was incubated at 37°C with *Mse*I [New England Biolabs (NEB), Ipswich, MA, United States], T4 DNA ligase (NEB), ATP (NEB), and a *Mse*I Y-adapter N containing barcode. Restriction-ligation reactions were heat-inactivated at 65°C, and then digested by additional restriction enzymes *Nla*III and *EcoR*I at 37°C. For the SE RIL population, the *Rsa*I enzyme was used to digest the genomic DNA. A single nucleotide (A) overhang was subsequently added to the digested fragments using a Klenow fragment (3′→5′ exo^-^) (NEB) and dATP at 37°C. Duplex tag-labeled sequencing adapters (PAGE-purified, Life Technologies, Wilmington, DE, United States) were then ligated to the A-tailed fragments using T4 DNA ligase.

Polymerase chain reaction (PCR) was performed using diluted restriction ligation DNA samples, dNTPs, Q5^®^ High-Fidelity DNA polymerase, and PCR primers. The PCR products were purified using Agencourt AMPure XP (Beckman, Irvine, CA, United States) and pooled, then separated by 2% agarose gel electrophoresis. Fragments that were 375- to 400-bp (with indexes and adaptors) in size were isolated using a gel extraction kit (Qiagen). These fragment products were then purified using Agencourt AMPure XP (Beckman, Irvine, CA, United States) and then diluted for sequencing. Then, pair-end sequencing (each end was 125 bp in length) was performed on an Illumina HiSeq 2500 system (Illumina, Inc., San Diego, CA, United States) according to the manufacturer’s recommendations.

### Sequence Data Grouping and SNP Identification

The sequences of each sample were sorted according to the barcodes. To ensure that the reads were reliable and without artificial bias (low-quality paired reads, which mainly resulted from base-calling duplicates and adapter contamination) in the following analyses, raw data (raw reads) were first processed through a series of quality control (QC) procedures using in-house C programs. The QC standards included removal of the following: (1) reads with ≥10% unidentified nucleotides (N); (2) reads with >50% bases having Phred quality < 5; (3) reads with >10 nt aligned to the adapter, which allow ≤10% mismatches; and (4) reads that contain *Mse*I, *Nla*III, *EcoR*I, or *Rsa*I cut-site remnant sequences. The Burrows-Wheeler Aligner (BWA v0.7.10) (Li and Durbin, 2009) was used to align the clean reads of each sample against the reference genome (settings: mem -t 4 -k 32 -M -R), where -t is the number of threads, -k is the minimum seed length, -M is an option used to mark shorter split alignment hits as secondary alignments, and -R is the read group header line. Alignment files were converted to BAM files using SAMtools software (v1.6) (Li et al., 2009). If multiple read pairs have identical external coordinates, then only the pair with the highest mapping quality was retained. Variant calling was performed for all samples using the GATK (v3.0-0-g6bad1c6) (Wang et al., 2010) software. SNPs were filtered using a Perl script. ANNOVAR (v20170716) (Wang et al., 2010) was used to annotate SNPs based on the GFF files of the reference genome. Parent polymorphic markers were classified into eight segregation patterns (ab × cd, ef × eg, hk × hk, lm × ll, nn × np, aa × bb, ab × cc, and cc × ab). The aa × bb type is suitable for inbreeding groups [F_2_ (the result of selfing the F_1_ of a cross between two fully homozygous diploid parents), RILs, doubled haploid (DH) populations: the result of doubling the gametes of a single heterozygous diploid individual)], and the remaining markers were applied to the hybrid groups [e.g., CP (a population resulting from a cross between two heterogeneously heterozygous and homozygous diploid parents)].

The aa × bb segregation pattern markers were then selected for genetic linkage map construction of two RIL populations. Prior to the map construction, markers with segregation distortion (*p* < 0.01), markers with >30% missing genotype data (the threshold of missing ratio was set to 40% for chromosome 11 of the SE population to ensure that molecular markers were evenly distributed), or containing abnormal bases were filtered.

### Map Construction

Chi-square (χ^2^) tests were conducted for all SNPs to detect segregation distortion. For bin mapping, markers with the same genotype were divided into bin markers using a Perl script. Based on physical position, the markers were divided into 20 linkage groups (or chromosomes), and then HighMap software (Liu et al., 2014) was used to order the markers in every linkage group. A total of 5,255 SNP markers and 2,063 bin markers were detected for the SE and DW populations, respectively. Compared to the DW population, SE had more markers and relatively higher genotype data losing ratio (the proportion of unsuccessfully genotyped individuals at a molecular marker locus). Despite incomplete genotypic data, the data integrity of all markers remained high.

### QTL Analysis Using High-Density Genetic Maps

Quantitative trait loci for flowering time, maturity, and reproduction period in different environments were detected by multiple-QTL model (MQM) mapping using the MapQTL5 package (Van Ooijen, 2004). Windows QTL Cartographer 2.5 (WinQTLCart 2.5) (Wang et al., 2001) was also employed to identify QTLs by composite interval mapping (CIM) method (Zeng, 1994). The LOD threshold for declaring significant QTLs included the QTLs across environments and the average data of three traits in different environments that was calculated using a permutation test (PT) at a significance level of *P* < 0.05, *n* = 1,000. To compare the results with the QTLs detected in previous studies with a lower criterion (lower LOD scores), non-significant QTLs with a LOD score of >2.5 were also included in the analysis. LOD score values between 2.5 and the permutation test LOD threshold were used to declare suggestive QTLs.

### Candidate Gene Identification

The markers in the confidence intervals of the major QTLs that can be steadily detected in different environments and by different methods of QTL analysis were selected to identify the candidate genes. The sequences of these markers were then mapped to the reference genome Gmax_275_Wm82.a2.v1 (Schmutz et al., 2010) in Phytozome database. Based on the position of these flanking markers, all the genes within the confidence interval were identified as candidate genes. To show the confidence intervals of the map positions of each QTL, one-LOD and two-LOD support intervals (Lander and Botstein, 1989) were constructed, in which the LOD values are less than one and two from the maximum, respectively. One-LOD support intervals were defined by the points on the genetic map at which the likelihood ratio decreased by a factor of 10 from the maximum (Lander and Botstein, 1989). In this study, the high confidence interval for each QTL was assigned as a 1.5-LOD drop relative to the peak LOD (Zhou et al., 2016). The candidate genes in the high-confidence interval of the major QTLs were categorized using Gene Ontology (GO) analysis. The filtered working gene list of the soybean genome was downloaded from Phytozome^1^ to identify possible candidate genes within each QTL confidence interval. We analyzed SNPs and Insertion/deletions (Indels) between parents. In addition, the accurate position of these SNPs and Indels were determined to assess whether these lead to amino acid substitutions. We selected the most likely candidate genes within the confidence interval by testing for either associations with gene functions or associations between the gene and the pathways in which the phenotype is involved.

Nucleotide sequence polymorphisms between parents were analyzed using the following methods. The details on the resequencing data of the parents are shown in Supplementary Table 1. SNPs were called from the re-sequencing data of the parents. Paired-end resequencing reads were mapped to the Williams 82 soybean reference genome sequence (Gmax_275_Wm82.a2.v1; Schmutz et al., 2010) using BWA (v0.7.10) (Li and Durbin, 2009) using default parameters. SAMtools (v1.6) (Li et al., 2009) was used to convert mapping results into a binary alignment/map (BAM) format, then the resulting BAM files were sorted based on chromosomal positions of the SNPs. Duplicated reads were filtered using the Picard package (v1.90)^2^. The GATK software (v3.0-0-g6bad1c6) (McKenna et al., 2010) was used to realign the reads around Indels and produce a realigned BAM file for each accession as follows: the Realigner Target Creator tool (McKenna et al., 2010) was used to identify regions where realignment was needed, and then the Indel Realigner tool was used to realign these regions. SNPs with quality scores of <40 were discarded. Five software programs were used to detect Indels, namely, SAMtools (v1.7) (Li, 2011), GATK software (v3.0-0-g6bad1c6) (McKenna et al., 2010), Varscan (v1.0) (Koboldt et al., 2009), Pindel (v1.0) (Ye et al., 2009), and Soapindel (v2.1) (Li et al., 2013). Compared to SNP calling, Indel calling is more difficult. Calling Indels from the mapping of short paired-end sequences to a reference genome is much more challenging than SNP calling because the indel itself interferes with accurate mapping (Li et al., 2008; Li and Durbin, 2009). Furthermore, false-positive SNPs may occur around Indels and influence the accuracy of the Indel calling (Li et al., 2013). The powerful Indel calling approach has a low false-positive rate for long Indels. This will provide more reliable information for the identification of candidate genes and subsequent efforts in fine mapping. To optimize the Indels, we trained the Support Vector Machine (SVM) filter by simulative data and filtered the Indels using the SVM filter. We used the libsvm software package (Chang and Lin, 2011) for the application of SVM. High-quality Indels among parents were selected for further analysis.

The candidate genes in the interval of two major QTLs on chromosomes 4 and 6 were categorized using GO analysis. Blast2GO 4.0 (BioBam Bioinformatics S.L. Valencia, Spain) or the Phytozome database was used to determine the GO ID of candidate genes. Then, WEGO 2.0 (Ye et al., 2006) was used for visualization, comparing, and plotting the results of GO annotation. The process for obtaining the GO ID using Blast2GO- was as follows: First, CDS sequences were downloaded from the Phytozome database in FASTA format. Second, the sequences were aligned to the Nr database of NCBI for Nr annotation. Then, the Nr annotation result was converted to a trusted GO annotation using the Blast2GO database to obtain the GO ID. Default parameter settings were employed.

## Results

### Development of Two RIL Populations

**FIGURE 1:**
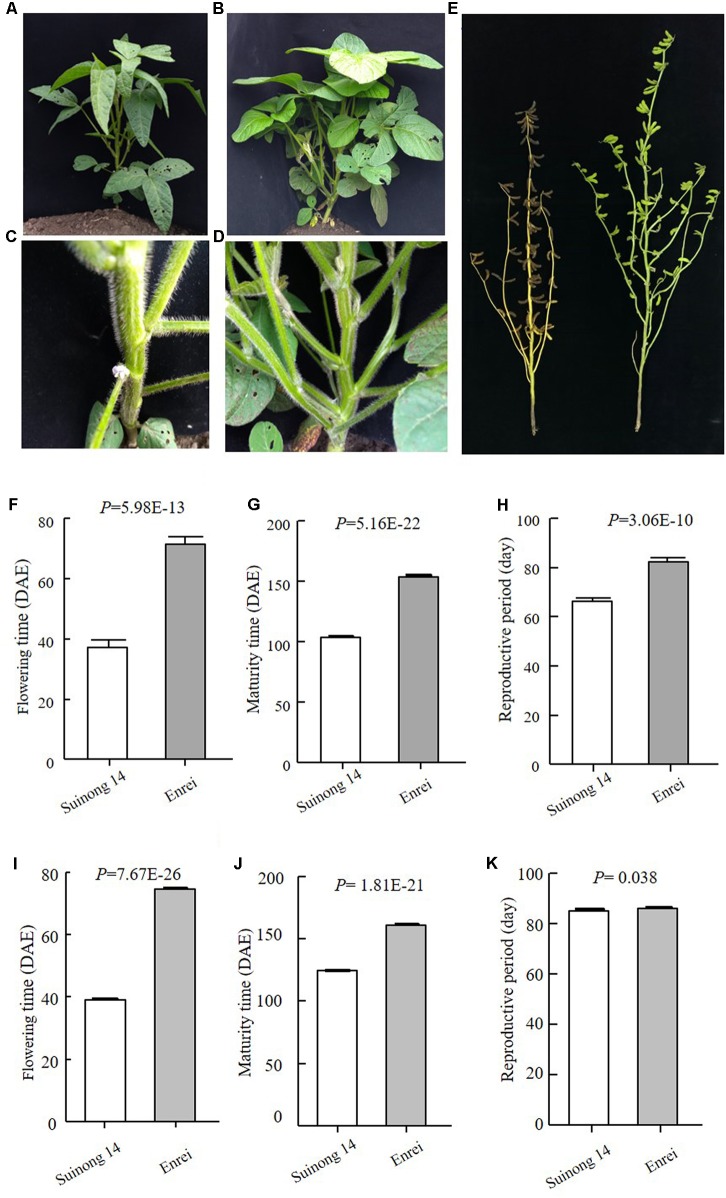
Phenotypes of the parents of SE population. **(A–D)** Plants when Suinong 14 had flowered, while Enrei was still in the vegetative growth stage. (**A,C** Suinong 14; **B,D** Enrei). **(E)**. Plants when Suinong 14 has already fully matured, while Enrei was still in the seed filling growth stage. Left is Suinong 14 and right is Enrei. **(F–H)** Phenotypes data of the parents in 2016, Harbin. **(I–K)** Phenotypes data of the parents in 2017, Harbin. DAE, day after emergence.

**FIGURE 2:**
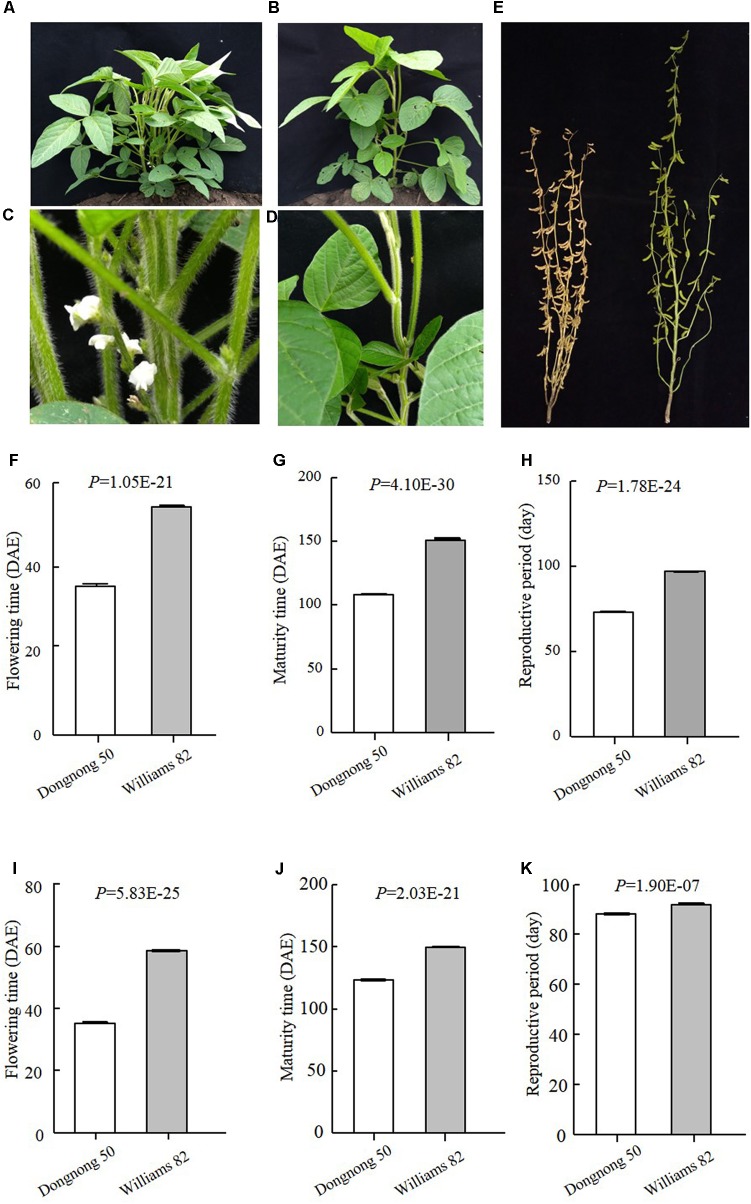
Phenotypes of the parents of DW population. **(A–D)** Plants when Dongnong 50 had flowered, while Williams 82 was still in the vegetative growth stage. (**A,C** Dongnong 50; **B,D** Williams 82). **(E)** Plants when Dongnong 50 has already fully matured, while Williams 82 was still in the seed filling growth stage. Left is Dongnong 50 and right is Williams 82. **(F–H)** Phenotypes data of the parents in 2016 Harbin. **(I–K)** Phenotypes of the Parents in 2017, Harbin. DAE, day after emergence.

Early maturity is critical for soybean to adapt to high latitudes and to reach successful grain yield harvest before frosting occurs. Identification of novel QTLs or genes is of great interest to understand soybean adaptation to high latitudes and molecular breeding. Suinong 14 is an elite cultivar in Northeast China that exhibits early flowering and maturity (**Figure 1**), whereas Enrei is an elite cultivar developed in the central region of Japan (Nagano prefecture) that shows very late flowering and late maturity (**Figure 1**). Dongnong 50 is another cultivar developed in Northeast China, and Williams 82 is the soybean cultivar from Northern America and its genome sequence has been used as reference. However, the flowering time and maturity between Dongnong 50 and Williams 82 largely differ (**Figure 2**). Due to significant variations in flowering time and maturity between the parents (**Figures 1**, **2**), we assumed that there are novel QTLs or genes in these two RIL populations. Therefore, we conducted NGS to construct high-density genetic linkage maps and QTL identification for flowering time and maturity in the SE and DW RIL populations.

### Analysis of Sequencing Data

The parents were resequenced at a higher coverage level to enhance the chances of detecting more SNP markers. The reference genome Gmax_275_Wm82.a2.v1 comprised 955,380,172 bp of assembled and anchored sequences. The average sequencing depth of the parents Suinong 14, Enrei, Dongnong 50, and Williams 82 in this study was 12.1×, 14.5×, 16.23×, and 12.6×, respectively. For Suinong 14 and Enrei, a total of 43,711,421 and 52,956,053 reads were respectively generated by sequencing the two parents. Of the total reads, 98.38 and 98.21% of the reads were mapped to the reference genome, and a total of 13,113,426,300 and 15,886,815,900 bases were identified in Suinong 14 and Enrei, respectively. Only reads aligned to unique positions on the reference genome were retained for subsequent SNP calling and genotyping. After data filtering, 90.66 and 89.56% of the reads were of high quality, with a Q30 ratio and GC content of 35.73 and 35.73%, respectively (Supplementary Table 1). Approximately 159,017 polymorphic SNPs were identified between Suinong 14 and Enrei. For Dongnong 50 and Williams 82, 89.18% and 85.98% of the total reads were of high quality, with an average Q30 ratio and GC content of 39.55 and 40.41%, respectively. The proportion of reads mapped to the reference genome was 97.34 and 95.80%, respectively. A total of 18,045,843,300 and 13,605,185,700 bases were identified, respectively (Supplementary Table 1). A total of 2,051,590 polymorphic SNPs were validated between Dongnong 50 and Williams 82 and used in linkage map construction.

In the RILs, SNPs segregate in a 1:1 ratio. After filtering out SNPs exhibiting significant segregation distortion (*p* < 0.001, χ^2^ test), a total of 88,664 and 2,021,590 SNPs were retained as assessed whether these could be utilized as markers. Because the parents are homozygous inbred lines, for Suinong 14 and Enrei, 81,221 homozygous polymorphic SNPs were selected. For Dongnong 50 and Williams 82, 1,285,743 homozygous polymorphic SNPs were identified.

### Construction of Genetic Linkage Maps

**FIGURE 3:**
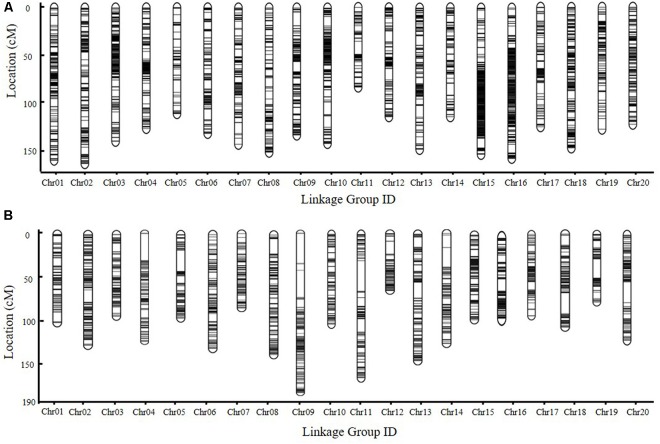
The genetic map of RIL populations. **(A,B)** Genetic map of SE and DW RIL population constructed with HighMap software, respectively. Chr., chromosome.

For the SE RIL population, polymorphic SNPs mapped to the same position were defined as one SLAF locus. For the DW RIL population, adjacent 100-kb intervals with the same genotype across the entire RIL population were considered as a single recombination bin locus (Huang et al., 2009; Davey et al., 2013). The chi-square test was performed to assess segregation distortion. Markers with significant segregation distortion were initially excluded from map construction. A total of 5,255 SLAF markers (Supplementary Table 2) and 2,063 bin markers (Supplementary Table 3) were identified in the SE and DW populations, respectively, which indicated that the majority of recombination events could be captured in the RIL populations. These markers were used in genetic map construction. In the SE genetic map, 5,255 SLAF markers fell within 20 linkage groups (LGs), and the genetic length was 2,756.17 M, with an average marker interval of 0.6045 cM (**Figure 3A** and Supplementary Tables 2, 13). In the DW map, 2,063 bin markers fell within 20 LGs; the genetic length was 2,458.55 cM, with an average marker interval distance of 1.203 cM (**Figure 3B**, and Supplementary Tables 3, 15). A relatively high collinearity was observed between the 20 LGs and the reference genome (Supplementary Figure 1), making the annotation of genes within QTL intervals feasible.

### Phenotypic Variations

The traits of flowering time (R1), maturity (R8), and RP of individuals from the two RIL populations and the parents were recorded in different environments and years. R1 were recorded during three growth seasons (Harbin in 2016 and 2017 and Mudanjiang in 2016), whereas R8 and RP were only recorded during two growth seasons (Harbin in 2016 and 2017). The results showed that Suinong 14 fl earlier than Enrei in different environments and years (**Figure 1** and **Table 1**), whereas Dongnong 50 flowered about 20 days earlier than Williams 82 in the two environments (**Figure 2** and **Table 2**). One-way ANOVA indicated *p*-values of <0.05, suggesting significant variations among the three traits between the two parents in different environments (**Tables 1**, **2**). Phenotypic variations (PV) involving the three traits were also observed in the RIL populations across different environments. The absolute value of skewness of the mean value of the traits in the two RIL populations across different environments was <1, indicating an approximately normal distribution. In different environments, either a positive or the negative transgressive segregation of the three traits in the two RIL populations was observed.

**Table 1:** Statistical analysis result of the parents and the whole SE population.

		Parents				RILs						
			
Trait	Environment	SN14	Enrei	Range	*P*-value	Min	Max	Range	Mean	CV (%)	Skewness	Kurtosis
R1	Harbin, 2016	37	72	34	5.98E-13	33	71	38	51.71	21.93	-0.17	-1.42
R1	Mudanjiang, 2016	#	#	#	#	38	73	35	54.44	15.72	-0.26	-0.83
R1	Harbin, 2017	39	75	36	7.67E-26	37	76	39	55.49	14.94	-0.11	0.13
R8	Harbin, 2016	104	154	50	5.16E-22	101	153	52	131.22	9.7	-0.59	-0.27
R8	Harbin, 2017	124	161(a)	37	1.81E-21	123	151	28	137	4.04	0.05	0.02
RP	Harbin, 2016	66	82	16	3.06E-10	56	115	59	79.59	15.32	0.85	0.23
RP	Harbin, 2017	85	86	1	0.038	67	103	36	82	9.43	1.02	0.90
Average R1		38	73	35	3.03E-32	37	73	36	53	16.69	-0.14	-0.95
Average R8		115	158	43	2.18E-17	113	152	39	134	6.52	-0.46	-0.21
Average RP		77	85	8	0.003	64	107	43	82	11.6	1.12	0.65


*SN14 is the abbreviation of Suinong 14, # represents the missing data and (a) represents Enrei could not normally mature in low temperature. R1: flowering time trait that means days from emergence to first open flower appeared on 50% of the plants in one line. R8: maturity time trait that means days from emergence to 95% of pods have turned their mature color on 50% of the plants in one line. RP: represents reproduction period and RP = R1 – R8. Harbin, 2016: the data of year 2016 in Harbin. Mudanjiang, 2016: the data of year 2016 in Mudanjiang. Harbin, 2017: the data of year 2017 in Harbin. Average R1: the average data of R1 in three environments (2016 and 2017 in Harbin, 2016 in Mudanjiang). Average R8: the average data of R8 in two environments (2016 and 2017 in Harbin). Average RP: the average data of RP in two environments (2016 and 2017 in Harbin). Min represents the minimum value of the population. Max represents the maximum value of the population. Range = Max - min. Mean: the average data of the whole population. CV (%): coefficient of variance in percentage type. The same as below.*

**Table 2:** Statistical analysis result of the parents and the whole DW population.

		Parents				RILs						
			
Trait	Environment	DN50	W82	Range	*P*-value	Min	Max	Range	Mean	CV (%)	Skewness	Kurtosis
R1	Harbin, 2016	35	54	19	1.05E-21	29	53	24	37.64	15.16	0.95	0.04
R1	Mudanjiang, 2016	#	#	#	#	36	58	22	45.63	13.19	0.08	-1.27
R1	Harbin, 2017	35	59	23	5.83E-25	35	59	24	44.10	16.07	0.64	-1.05
R8	Harbin, 2016	108	151	43	4.10E-30	103	144	41	122.92	8.66	-0.35	-0.93
R8	Harbin, 2017	123	150(b)	27	2.03E-21	121	146	25	133.29	3.55	0.24	-0.38
RP	Harbin, 2016	73	97	24	1.78E-24	67	103	36	85.35	9.51	-0.43	-0.44
RP	Harbin, 2017	88	92	4	1.90E-07	77	101	24	89.08	6.34	-0.02	-0.80
Average R1		35	56	21	2.44E-32	35	57	22	42.64	13.44	0.68	-0.67
Average R8		116	151	35	7.22E-22	114	142	28	128.57	5.63	-0.18	-0.98
Average RP		81	94	13	4.78E-09	75	100	25	87.33	6.29	0.02	-0.56


*DN50, Dongnong 50; W82, Williams 82; #presents the missing data and (b) presents Williams 82 could not mature normally under low temperature.*

Two-way ANOVA revealed significant differences among environments and lines in both RILs (**Table 3**). Phenotypes were affected by both genotype and environment. Environmental effects contributed more to the total PVs than line effects, suggesting that there was a common photoperiod response in R1, R8, and RP. The *h*^2^_b_ of the three growth duration-related traits in the SE and DW populations ranged from 0.77 to 0.99 and 0.55 to 0.88, respectively, among different environments. The estimated values of *h*^2^_b_ of corresponding traits in the SE population were higher than that of DW. The R1 and RP traits were observed with relatively higher *h*^2^_b_ values than R8 in both RIL populations, suggesting that the R1 and RP traits are mainly controlled by genetic factors (**Table 3**).

**Table 3:** Analysis of variance for three traits of two RILs in different environments.

RIL	Trait	Source of variation	DF	MS	*F*-value	*P*-value	*h*^2^_b_
SE	R1	Line	139	229.25	11.48	4.18E-64	0.97
		Environment	2	475.32	23.81	2.92E-10	
		Error	274	19.96			
		Total	416				
	R8	Line	139	153.07	3.88	7.58E-15	0.77
		Environment	1	2138.28	54.25	1.46E-11	
		Error	138	39.41			
		Total	279				
	RP	Line	139	167.74	4.23	4.22E-16	0.99
		Environment	1	313.63	7.91	5.70E-03	
		Error	134	39.66			
		Total	275				
DW	R1	Line	125	96.52	7.19	8.90E-40	0.71
		Environment	2	2260.32	168.45	5.41E-47	
		Error	249	13.42			
		Total	377				
	R8	Line	125	99.57	2.98	1.95E-09	0.52
		Environment	1	6455.02	193.36	7.54E-27	
		Error	121	33.38			
		Total	248				
	RP	Line	125	57.46	1.46	0.02	0.88
		Environment	1	836.78	21.20	1.04E-05	
		Error	120	39.48			
		Total	247				


*DF, degrees of freedom; MS, mean square; *h^2^_*b*_*, broad sense heritability.*

We then calculated the correlation coefficients among R1, R8, and RP from the two RIL populations in the two environments (Harbin in 2016 and 2017) and the average value of the three traits in the two environments. For the SE RIL population, positive correlation coefficients were observed between R1 and R8 and between RP and R8, whereas a negative correlation was detected between R1 and RP (Supplementary Figure 2 and Supplementary Table 4). Similar correlations were also observed among R1, RP, and R8 in the DW RIL population (Supplementary Figure 3 and Supplementary Table 4). These results suggest that maturity consists of flowering time and RP, and a balance between appropriate flowering time and RP is critical to maximize the maturity and yield productivity during short growth periods at high latitudes. In addition, different environmental conditions also influence maturity. Genetic and environmental interactions should thus be taken into the consideration to elucidate the underlying mechanism of flowering time and maturity.

### QTL Mapping for Flowering Time, Maturity, and Reproductive Period

Next, we conducted QTL identification using the phenotypic data across three environments in the two RIL populations. Due to environmental effects, the QTLs for the three traits were assessed separately in each environment, and the average values of different environments were also analyzed. Common loci among multiple environments were considered as consistent QTLs. The threshold of the LOD scores for evaluating the statistical significance of QTL effects is shown in Supplementary Table 5.

**FIGURE 4:**
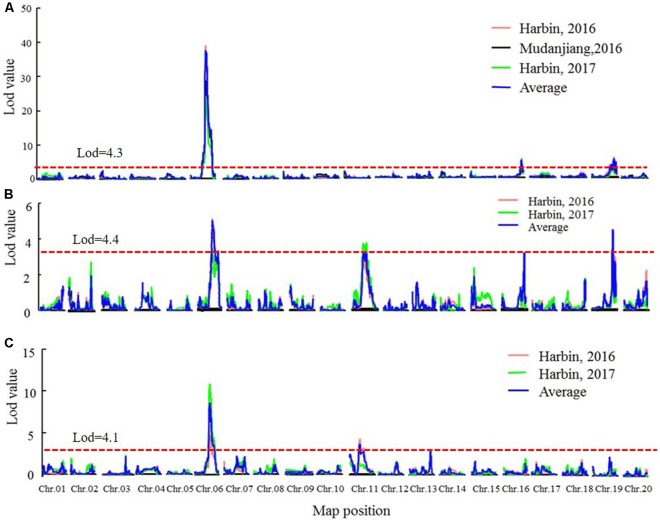
The LOD value of the QTLs detected in SE RIL population under different environments and QTLs detected using the average data of different environments (MQM method). **(A)** QTLs of Flowering time trait (R1). **(B)** QTLs of maturity time trait (R8). **(C)** QTLs of reproductive period trait (RP). Harbin, 2016: The data of year 2016 in Harbin. Mudanjiang, 2016: The data of year 2016 in Mudanjiang. Harbin, 2017: The data of year 2017 in Harbin. Average: the average data in different environments. Chr., chromosome.

Quantitative trait loci analysis using the MQM and CIM methods (**Table 4**, **Figure 4**, Supplementary Figure 4, and Supplementary Table 7) indicated that in the SE RIL population, a QTL for the R1 trait, namely, *qR1-C2*, was consistently detected across the three environments (Harbin in 2016, Mudanjiang in 2016, and Harbin in 2017), and as expected, the QTL was also detected using the average data of the three environments (**Table 4** and **Figure 4**). In this study, compared to the MQM method, CIM imparts similar detection results on the major QTL, CIM had relatively larger LOD values and smaller QTL intervals. The overlapping interval from Marker 381516 to Marker 400193 between the two methods was defined as the final QTL interval of the R1 trait on chromosome 6. QTL *qR1-C2* spanned a genetic distance encompassing 77.06 cM to 96.86 cM, -at physical positions of 15,588,950–45,035,341 to the reference genome and could explain 43.5–78.9% of the observed PV (**Table 4**). The phenotypes of RP and R8 traits were surveyed only in two environments (Harbin in 2016 and Harbin in 2017). The QTL named *qRP-C2* for the RP traits were detected in both growth seasons as well as using the average data and was located between Markers 386215 and 408436 on chromosome 6, at physical positions of 28,154,587–46,314,802 to the reference genome, and explained 14.2–30.8% of the observed PV using the MQM method (**Table 4**). The QTLs of the RP traits on chromosome 6 that were detected using the CIM method were situated within the same QTL interval for the R1 trait and explained 5.06–39.4% of the observed PV (**Table 4** and Supplementary Table 7). QTL intervals of R8 traits on chromosome 6 overlapped with those that of the R1 and RP traits. QTL *qR8-C2* explained 9.7–23.4% of the observed PV (**Table 4** and Supplementary Table 7). We conclude that the QTLs, namely, *qR1-C2, qRP-C2*, and *qR8-C2*, which were mapped to chromosome 6, are the same QTLs simultaneously conditioning R1, RP, and R8 and are the major QTLs in the SE RIL population. Other minor QTLs (Supplementary Table 7) were also detected using the two QTL analysis methods. The QTL *qRP-B1* was detected only in Harbin in 2016 using the average data of the two growth seasons, was located within the interval encompassing Markers 648557 to 670885 on chromosome 11, at physical position of 5,106,306–28,115,520 relative to the reference genome, and explained 10.3–20.9% of the observed PV. This interval could also be detected in the R8 trait. *qR1-J* was located within the interval encompassing Markers 1081647 to 1097552 on chromosome 16 at physical positions of 30,795,613–35,841,366 and explained 2.5–17.1% of the PV. These results suggest that maturity traits (R8) are not affected by either flowering time (R1) or RP alone, but both, which makes the genetic dissection of maturity traits more complicated. There might also be different molecular mechanisms regulating pre-flowering and post-flowering responses. Molecular cloning of the candidate genes of these QTLs and dissection of the functional interactions of these genes facilitate in elucidating the gene regulatory networks underlying soybean flowering and maturity.

**Table 4:** Detail information about the stable QTLs in SE population.

Method	QTL name	Environment	Chr.	Flanking markers	Interval (cM)	Physical length	Max LOD	PVE (%)	ADD
MQM	*qR1-C2*	Harbin, 2016	6	Marker379905–Marker409386	70.59–108.81	14,777,378–47,223,902	38.66	78.9	10.06
	*qR1-C2*	Mudanjiang, 2016	6	Marker380087–Marker409386	71.73–108.81	14,823,291–47,223,902	28.44	66.3	6.95
	*qR1-C2*	Harbin, 2017	6	Marker380527–Marker409386	72.83–108.81	14,924,591–47,223,902	23.09	54.2	6.08
	*qR1-C2*	Average	6	Marker379905–Marker409386	70.59–108.81	14,777,378–47,223,902	37.28	72.6	7.51
	*qR8-C2*	Harbin, 2016	6	Marker386215–Marker393195	79.65–87.72	28,154,587–38,814,913	4.98	18.5	5.46
	*qR8-C2*	Average	6	Marker386215–Marker393195	79.65–87.72	28,154,587–38,814,913	4.88	18.1	3.72
	*qRP-C2*	Harbin,2016	6	Marker386215–Marker393195	87.36–87.72	28,154,587–38,814,913	4.65	14.2	-4.58
	*qRP-C2*	Harbin, 2017	6	Marker386215–Marker408436	87.36–102.77	28,154,587–46,314,802	10.78	30.8	-4.26
	*qRP-C2*	Average	6	Marker386215–Marker398081	87.36–94.08	28,154,587–44,645,578	8.55	25.3	-4.76
CIM	*qR1-C2*	Harbin, 2016	6	Marker381516–Marker400193	77.06–96.89	15,588,950–45,035,341	41.91	70.2	9.63
	*qR1-C2*	Mudanjiang, 2016	6	Marker381516–Marker400193	77.06–96.89	15,588,950–45,035,341	37.11	72.2	7.60
	*qR1-C2*	Harbin, 2017	6	Marker381516–Marker400193	77.06–96.89	15,588,950–45,035,341	26.06	43.5	5.88
	*qR1-C2*	Average	6	Marker381516–Marker400193	77.06–96.89	15,588,950–45,035,341	45.91	73.5	7.91
	*qR8-C2*	Harbin, 2016	6	Marker381516–Marker400193	77.06–96.89	15,588,950–45,035,341	8.18	23.4	6.18
	*qRP-C2*	Harbin, 2017	6	Marker381872–Marker400193	77.66–96.89	15,741,239–45,035,341	17.95	39.2	-5.09
	*qRP-C2*	Average	6	Marker381872–Marker400193	79.66–96.89	15,741,239–45,035,341	11.33	23.0	-4.64


*MQM, multiple-QTL model; CIM, composite interval mapping; PVE (%), percentage of phenotypic variance explained by the QTL; ADD, The additive effects contributed by QTLs. We used letter A represented markers with paternal (Enrei) genotype, and letter B represented markers with maternal (Suinong14) genotype in QTL analysis. So positive value (+) of the additive effect indicates the allele from Enrie enhance phenotype, negative value (-) of the additive effect indicates the allele originating from Suinong 14 reduced phenotype; Max LOD, maximum logarithm-of-odds (LOD) scores.*

**FIGURE 5:**
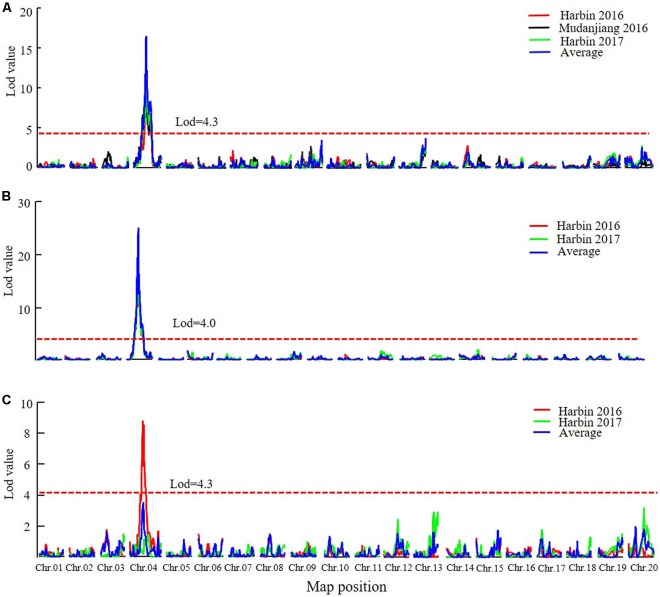
The LOD value of the QTLs detected in DW RIL population under different environments and QTLs detected using the average data of different environments (MQM method). **(A)** QTLs of Flowering time trait (R1). **(B)** QTLs of maturity time trait (R8). **(C)** QTLs of reproductive period trait (RP). Harbin, 2016: The data of year 2016 in Harbin. Mudanjiang, 2016: The data of year 2016 in Mudanjiang. Harbin, 2017: The data of year 2017 in Harbin. Average: the average data in different environments. Chr., chromosome.

In the DW RIL population, QTLs of *qR1-C1*, *qRP-C1*, and *qR8-C1* that control the R1, RP, and R8 traits were all identified in the almost same position on chromosome 4 and were thus considered as one major QTL that simultaneously controls all three traits (**Figure 5**, **Table 5**, and Supplementary Figure 4). This QTL interval was flanked by markers Gm04_58 and Gm04_84, with a physical position ranging from 9,226,038 to 44,180,506 and influenced all the three traits in different environments and could be consistently detected, except for the reproduction trait in Harbin of 2017. These three QTLs explained 34.2–53.1%, 15.6–59.4%, and 33.7–59.9% of the PV of R1, RP, and R8, respectively. The minor QTLs detected with the two QTL analytical methods are presented in Supplementary Table 8. These results suggest that PVs in R1, RP, and R8 were mainly contributed by the major QTL on chromosome 4. Cloning the candidate gene conditioning this major QTL and investigating its functions in relation to photoperiod flowering and yield improvement at high latitude environments are thus warranted.

**Table 5:** Detail information about the stable QTLs in DW population.

Method	QTL name	Environment	Chr.	Flanking markers	Interval (cM)	Physical length	Max LOD	PVE (%)	ADD
MQM	*qR1-C1*	Harbin, 2016	4	Gm04_58-Gm04_88	52.84–73.70	9,226,038–45,726,081	12.56	36.8	-3.58
	*qR1-C1*	Mudanjiang, 2016	4	Gm04_47-Gm04_84	40.05–65.40	7,218,438–44,180,506	12.70	37.1	-3.71
	*qR1-C1*	Harbin, 2017	4	Gm04_47-Gm04_88	40.05–73.70	7,218,438–45,726,081	11.35	34.2	-4.20
	*qR1-C1*	Average	4	Gm04_47-Gm04_88	40.05–73.70	7,218,438–45,726,081	16.48	45.2	-3.84
	*qR8-C1*	Harbin,2016	4	Gm04_47-Gm04_89	40.05–85.69	7,218,438–45,997,654	19.12	51.4	-7.66
	*qR8-C1*	Harbin,2017	4	Gm04_48-Gm04_90	40.55–86.50	7,501,700–46,095,409	18.83	49.8	-3.34
	*qR8-C1*	Average	4	Gm04_47-Gm04_89	40.05–85.69	7,218,438–45,997,654	24.98	59.9	-5.60
	*qRP-C1*	Harbin,2016	4	Gm04_56-Gm04_84	48.67–65.40	8,793,441–44,180,506	8.51	27.5	-4.27
CIM	*qR1-C1*	Harbin,2016	4	Gm04_57-Gm04_84	49.89–65.40	9,118,002–44,180,506	18.95	39.6	-3.85
	*qR1-C1*	Mudanjiang,2016	4	Gm04_56-Gm04_84	48.68–65.40	8,793,441–44,180,506	15.78	36.3	-3.78
	*qR1-C1*	Harbin,2017	4	Gm04_55-Gm04_84	47.88–65.40	8,628,734–44,180,506	18.21	40.5	-4.66
	*qR1-C1*	Average	4	Gm04_56-Gm04_84	48.68–65.40	8,793,441–44,180,506	24.36	53.1	-7.93
	*qR8-C1*	Harbin,2016	4	Gm04_55-Gm04_84	47.88–65.40	8,628,734–44,180,506	23.14	46.1	-3.24
	*qR8-C1*	Harbin,2017	4	Gm04_55-Gm04_84	47.88–65.40	8,628,734–44,180,506	13.26	33.7	-4.81
	*qR8-C1*	Average	4	Gm04_55-Gm04_84	47.88–65.40	8,628,734–44,180,506	24.92	49.9	-4.16
	*qRP-C1*	Harbin,2016	4	Gm04_52-Gm04_82	43.77–63.79	8,036,188–44,045,265	31.44	59.4	-5.67
	*qRP-C1*	Average	4	Gm04_58-Gm04_62	52.85–58.48	9,226,038–11,334,980	6.63	15.6	-2.21


*ADD, the additive effects contributed by QTLs. Different from SE population, we used letter A represented markers with maternal (Dongnong 50) genotype and B represented markers with paternal (Williams 82) genotype in QTL analysis. So positive value (+) of the additive effect indicated the allele from Dongnong 50 reduced phenotype, negative value (-) of the additive effect indicated the allele originating from Williams 82 enhanced phenotype; Max LOD, maximum logarithm-of-odds (LOD) scores.*

**FIGURE 6:**
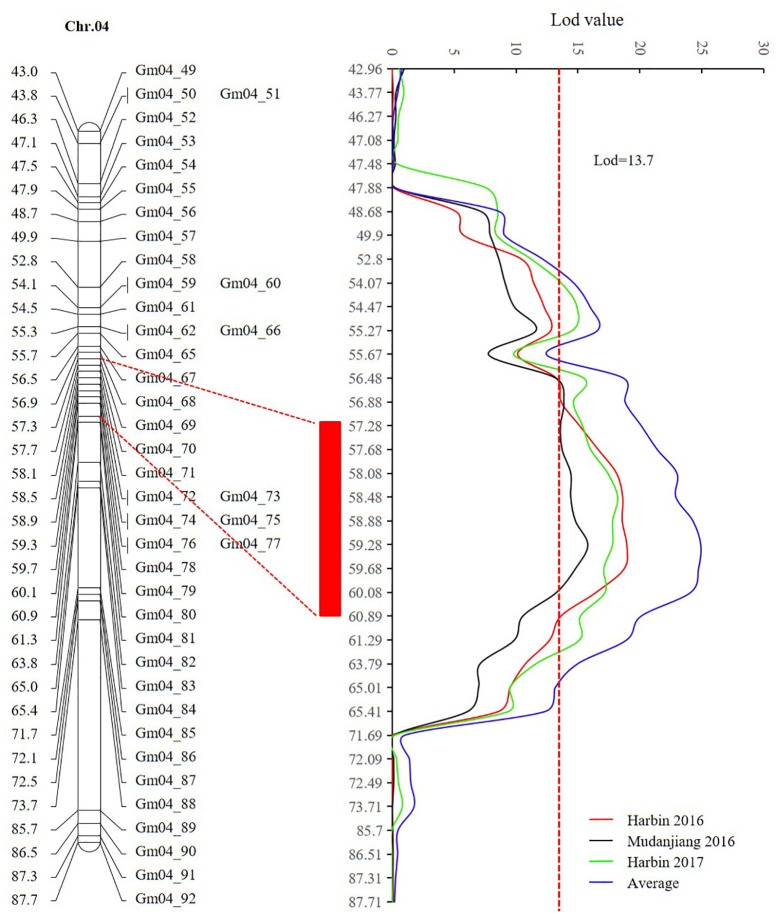
The high confidence interval of QTL detected on chromosome 4 (CIM method). The red rectangle represents the high confidence interval between markers Gm_69 and Gm_80 with physical positions from 13,212,370 to 43,843,500.

**FIGURE 7:**
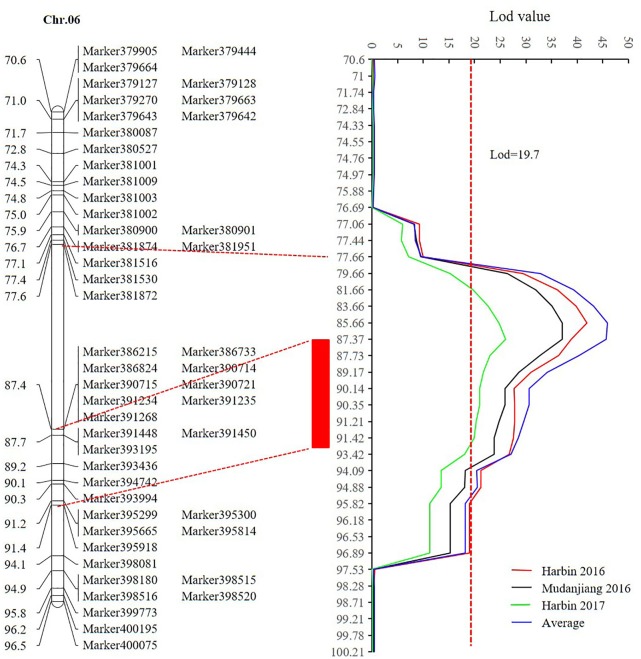
The high confidence interval of QTL detected on chromosome 6 (CIM method). Within the genetic interval from 77.66 cM to 87.4 cM between markers Marker381872 and 386215 with physical positions 15,741,239–28,154,637, no recombination was found according to the sequencing result. The red rectangle represents the high confidence interval between markers Marker386215 and 395918 with physical positions from 28,154,637 to 42,126,497.

Sequencing of the SE population did not detect any recombination events between markers 381872 and 386215, with physical positions encompassing 15,741,239 to 28,154,637 (**Figure 7** and Supplementary Table 14). Based on the soybean genome sequence (Schmutz et al., 2010), we determined that this interval is situated within a pericentromeric region, which generally features low recombination rates. In addition, with a large confidence interval, the position of one marker flanking the confidence interval was too far from that of the nearest marker within that confidence interval. The region between these two markers should thus be excluded from candidate gene identification. In this study, we used a 1.5-LOD drop on either side of the peak marker to delimit the QTL of the SE RIL population on chromosome 6. Thus, the final high confidence interval was between Markers 386215 and 395918, encompassing positions 28,154,637–42,126,497 (**Figure 7** and Supplementary Table 14). The high confidence interval of the major QTL in the DW population was between Gm04_69 and Gm04_80, at positions 13,212,370–43,843,500 (**Figure 6** and Supplementary Table 15).

### Candidate Gene Prediction

In this study, the major QTL of the SE population was detected on chromosome 6. Resequencing of the parents localized the major QTL within the physical interval of 14,777,428–47,231,089 and harbored 20,866 polymorphism SNPs and Indels between Suinong 14 and Enrei (**Figure 10B**). Genetic backgrounds of the parents in a region (∼20,000,000–30,000,000) surrounding centromere of chromosome 6 was relatively similar. Furthermore, fewer polymorphic loci were observed in the larger interval. The physical position of the peak LOD is about 28,154,587. The confidence interval of the flowering time QTL encompassed 15,742,176–42,126,497 and was assigned a 1.5-LOD drop relative to the peak LOD; a total of 389 SNPs and Indels were detected in genes (**Figure 10D** and Supplementary Table 11). Of these nucleotide variations, 99 SNPs/Indels were located within CDS regions, and 35 of these were synonymous mutations. The other 64 mutations are predicted to result in amino acid changes in 29 genes (**Figure 10D** and Supplementary Table 11), which were then classified as the most likely candidate genes.

The major QTL of the DW population was detected on chromosome 4, with a physical interval encompassing 9,622,245–44,284,689. According to the SNP and Indel calling results of resequencing the parents, there were 115,068 SNPs between Dongnong 50 and Williams 82 (**Figure 10A**). This high number of variations implies that the genetic background of the DW population significantly varies in this region of chromosome 4. Approximately 846 SNPs and Indels that result in amino acid changes were identified within the 1.5-LOD drop QTL interval, with positions encompassing 13,212,370–43,843,500, of which 194 polymorphic loci were shared between the DW and SE parents (**Figure 10C** and Supplementary Table 10). In the SE population, no QTLs of growth period related traits were detected on chromosome 4, suggesting that these 194 mutations may not lead to phenotypic differences in flowering time, maturity, and RP between Dongnong 50 and Williams 82. These variations can thus be preliminarily eliminated from candidate gene analysis. However, the DW and SE populations have different *E1* genotype backgrounds, and thus whether the same mutations function differently under the *E1* and *e1-as* genetic backgrounds requires further experimental verification. Other 652 polymorphic loci were only detected in the DW parents and not in the SE parents (**Figure 10D** and Supplementary Table 11).

**FIGURE 8:**
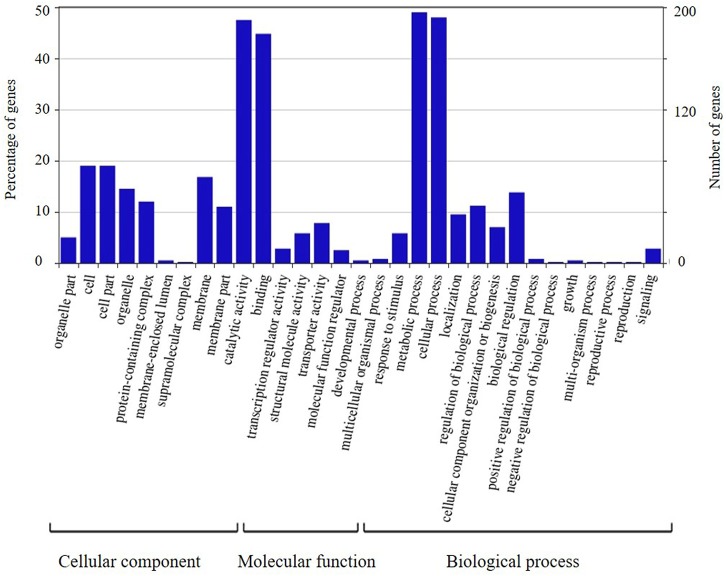
Annotation information of genes in the high confidence interval of QTL detected on chromosome 4 through Gene ontology (GO) analysis.

**FIGURE 9:**
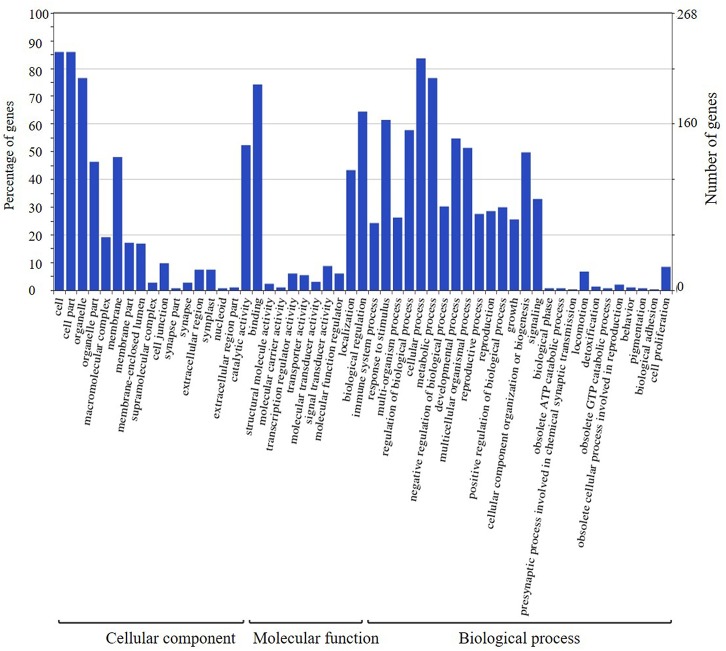
Annotation information of genes in the high confidence interval of QTL detected on chromosome 6 through Gene ontology (GO) analysis.

The candidate genes in the high-confidence interval of the two major QTLs in two populations were categorized using GO analysis (**Figures 8**, **9**). Within the high-confidence interval of the major QTL on chromosome 6, 298 genes could be functionally annotated using GO. Of these, 256, 264, and 265 were functionally annotated to the categories of cellular components and biological processes, respectively (Supplementary Table 17). Eighteen genes were related to transcription regulation activity, 20 genes were related to regulation of reproductive process, and 7 genes, namely, Glyma.06G238800, Glyma.06G239700, Glyma.06G241900, Glyma.06G242100.1, Glyma.06G242100.2, Glyma.06G242100.3, and Glyma.06G248100, were related to photoperiodism of flowering. Within the high-confidence interval of the major QTL on chromosome 4, 400 genes could be annotated using GO (Supplementary Table 16). Around 351, 132, and 268 of these were annotated to the functional categories of molecular, cellular component, and biological process, respectively. One gene, Glyma.04G139100.1, is related to the regulation of reproductive processes. Some reported orthologous genes related to photoperiod responses that regulate the flowering and reproduction in plants are listed in Supplementary Table 9. All these genes might be related to the traits assessed in the present study, but require further verification.

**FIGURE 10:**
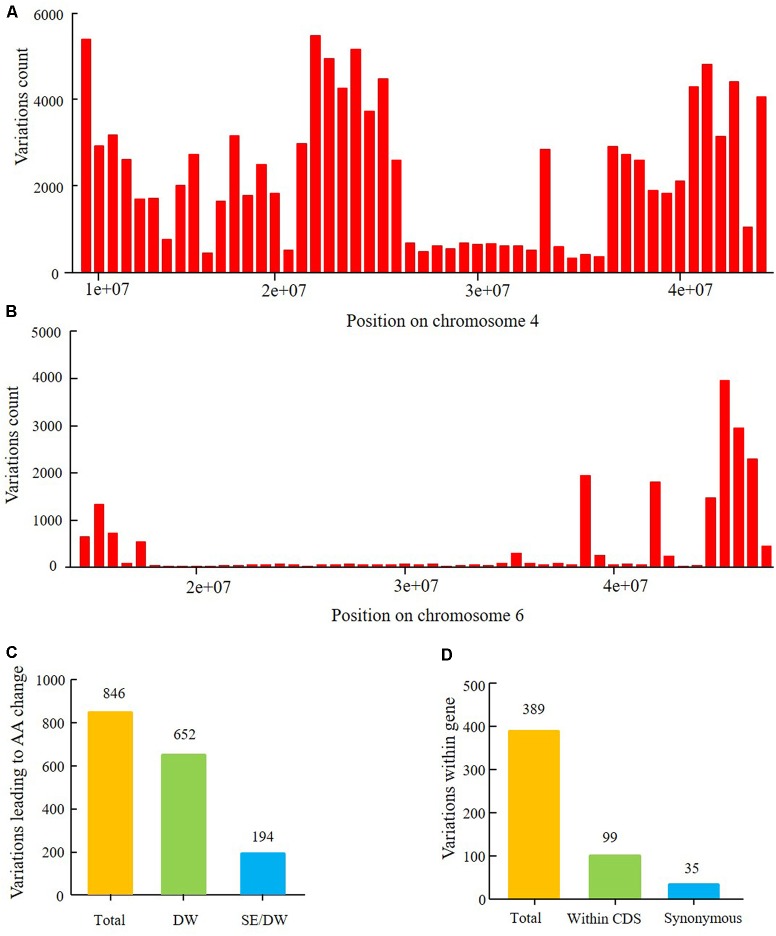
Nucleotide sequence polymorphism of parents of the two RIL populations within the intervals of two major QTLs. **(A)** Polymorphism SNP number between parents Dongnong 50 and Williams 82 within QTL interval from 9,226,038 to 44,284,689 on chromosome 4. **(B)** Polymorphism SNPs and Indels between Suinong 14 and Enrei within the QTL interval from 14,777,428 to 47,231,089 on chromosome 6. **(C)** Polymorphism SNPs and Indels leading to amino acids (AA) change in the 1.5-LOD drop QTL interval from 13,212,370 to 43,843,500 on chromosome 4 between Dongnong 50 and Williams 82. Total: all SNPs and Indels; SE/DW: the same polymorphism SNPs and Indels between four parents of the two RIL populations which were with the same variation position and variation type; DW: SNPs and Indels uniquely detected between Dongnong 50 and Williams 82. **(D)** Polymorphism SNPs and Indels within gene (5′UTR, CDS, 3′UTR) in the 1.5-LOD drop QTL interval from 15,741,239 to 42,126,497 of chromosome 6 between parents Suinong 14 and Enrei. Total: all SNPs and Indels; Within CDS: SNPs and Indels number within the CDS region of one gene; Synonymous: variations that resulted in no change in amino acids.

The QTL on chromosome 16 with the highest LOD score was located near Marker 1093929, with a physical position of 34,086,866. We analyzed sequence polymorphisms near Marker 1093929, with the 1.5-LOD drop on either side of the peak marker from positions 34,064,162–34,328,726 between Suinong 14 and Enrei. The interval encompassed 62 genes, of which 25 had polymorphism within the CDSs (Supplementary Table 12). These 25 genes might be the candidate genes of flowering time trait. The QTL on chromosome 19 with the highest LOD score was located near Marker 1304107 whose physical position was 45,085,367, whereas the *Dt1* gene *Glyma.19G194300 (TERMINAL FLOWER 1)* is located within the region. In addition, the parents harbored polymorphisms at the *Dt1* locus (Supplementary Figure 5), suggesting that the candidate gene for the QTL on chromosome 19 might be *Dt1*.

## Discussion

Legumes play critical roles in ensuring global food security and agricultural sustainability. Soybean is one of the most economically important plant oil and protein crops. Soybean is a short-day plant (SDP) and is highly sensitive to photoperiod and latitude. Incorporation of new genetic resources has enabled the gradual extension of commercial soybean cultivation toward higher latitudes (Cao et al., 2017). Absence of or low sensitivity to long-day photoperiod is necessary for short-day crops such as rice and soybean, to adapt to higher latitudes. Understanding the genetic diversity in flowering time, photoperiod insensitivity, and post-flowering photoperiodic responses may facilitate in improving final grain yield in specific regions (Xu et al., 2013).

Soybean undergoes different growth stages, starting from germination to maturity, when these are harvested. Flowering time is an important factor that affects the duration of the entire growth period of soybean. QTL analysis indicates that the QTL loci for R1 could also be detected in the R8 phenotype. The duration of post-flowering time is another important trait that influences growth rate, yield, and seed quality of soybean.

The maturity locus *E1* influences flowering time in soybean. The recessive allele *e1-as*, a non-synonymous substitution occurring in the putative nuclear localization signal, leads to loss of E1 protein localization specificity and represses earlier flowering (Xia et al., 2012). To eliminate the interference of the *E1* locus and find different flowering regulatory mechanisms under different *E1* backgrounds, we created two RIL populations with *E1* and *e1-as* backgrounds, respectively, and their *E2–E4* gene loci were exactly the same. In this study, Suinong 14 and Enrei both had dominant *E1* backgrounds, but Suinong 14 flowered about 35 days earlier and matured 50 days earlier than Enrei (**Table 1**). Dongnong 50 and Williams 82 were both recessive *e1- as*, but Williams 82 flowered 20 days late and matured about 50 days late (**Table 2**). These findings suggest that there are other genetic factors regulating flowering and maturation time in soybean that are influenced by genetic background. In the background of dominant *E1* cultivars, which still showed early flowering phenotypes, there might have been mutations in the upstream or downstream genes in the regulatory network of *E1* that eliminate the *E1* inhibitory effect on *FT* genes. In the *e1-as* background, which still flowered and matured late, flowering and maturation times might depend on other regulatory factors in a pathway that does not require *E1*. The factors might function similarly to *E1* to repress flowering and maturation in soybean. The four parents in this study were cultivars from different regions. Suinong 14 and Dongnong 50 were from China, Enrei was from Japan, and Williams 82 was from the United States. During breeding, different genotypes are selected, which in turn lead to differences in flowering time, maturity time, and other agronomic traits. SNP and Indels that may affect gene function provide a reservoir of novel genes and genetic variations for soybean improvement. Searching for polymorphisms that underlie variations in the agronomic traits of flowering and maturity and genes that exhibit a signature of artificial selection by breeders may help in the identification of candidate genes that play important roles in soybean domestication, diversification, and improvement.

Preliminary QTL mapping has established the approximate interval of QTLs for flowering time and maturity traits. Analysis of genetic backgrounds of the two RIL populations based on parental resequencing data provides more information on the candidate genes that may be used in fine mapping QTLs. In this study, we mapped two major flowering and maturity QTLs, one was mapped to chromosome 6 of the SE RIL population and the other to chromosome 4 of the DW RIL population. The major QTL *qR1-C2* of the SE RIL population that showed the highest LOD score is located near Marker 386215, with a physical position near 28,154,637. *E1* is a major maturity gene that largely influences flowering time and is located within the same genomic position (Xia et al., 2012). Therefore, we also analyzed the resequencing data of Suinong 14 and Enrei, including the upstream, CDS, and downstream *E1* genomic sequences. The results showed no sequence differences within the CDS and the 1.5-kb upstream fragment and no homozygous mutations within the 1.9-Kb downstream fragment of the parents, Suinong 14, and Enrei. Another maturity locus, *E7*, has been reported to control photoperiod sensitivity and is genetically linked to *E1* and *T* (Cober and Voldeng, 2001), which indicates that the QTL on chromosome 6 in this study might be *E7*. In addition, there are other reported QTLs that overlap with the interval identified in this study (Mansur et al., 1993; Orf et al., 1999; Funatsuki et al., 2005; Githiri et al., 2007; Liu and Abe, 2010; Oyoo et al., 2010; Sun et al., 2013; Hu et al., 2014; Zhang et al., 2015; Mao et al., 2017) (Supplementary Table 6). There may be several loci that affect multiple growth period-related traits such as time of first flowering, pod maturity, RP, and time to full maturity. Pleiotropic genes might affect multiple traits. The QTL intervals of *First flower 18-1*, *Pod maturity 21-1*, and *Pod maturity 25-1* (Chen et al., 2007; Palomeque et al., 2009) show almost similar high confidence intervals in our study.

The other major QTL *qR1-C1* in this study was mapped to chromosome 4 of the DW RIL population. The *qR1-C1* locus spanned marker Gm04_69 to Gm04_80, within the 1.5-LOD drop on either side of the peak. The delimitated interval was about 6.493 cM, with a physical position encompassing 13,212,370–43,843,500, whereas *E8* was mapped between markers Sat_404 and Satt136, with a physical position from 13,613,713 to 16,984,318 (Cober et al., 2010). From the mapping information, we assume that the major QTL *qR1-C1* might be controlled by *E8.* The QTL on chromosome 4 in this study also overlapped with other reported QTLs for reproductive stage lengths, pod maturity and total growth duration in Soybase^3^ and previous reports (Cheng et al., 2011; Rossi et al., 2013; Wang et al., 2015). These reported QTLs (Supplementary Table 6) and those detected in our study suggest that the major QTL *qR1-C1* plays very important roles in flowering, maturity, and the length of the RP in diverse genetic backgrounds.

The present study also detected several QTLs that separately controlling either flowering time or RP. For example, *qRP-B1* on chromosome 11 in the SE population was determined to play a role in RP traits but not in flowering time, whereas *qR1-J* on chromosome 16 and *qR1-L* on chromosome 19 is involved in flowering time. However, all of these loci affect time to full maturity. Both flowering time and the duration of the RP affect the final length of the whole growth period (**Tables 4**, **5**). These results may suggest that flowering time and RP are controlled by different genes and have relatively independent genetic mechanisms. The QTL *qRP-B1* on chromosome 11 in our study also overlapped with other reported QTLs for reproductive stage lengths and pod maturity (Lee et al., 1996, 2015; Zhang et al., 2004; Gai et al., 2007; Bachlava et al., 2009; Komatsu et al., 2012). All of these reported QTLs were mainly detected in reproductive development-related traits, which may suggest that *qRP-B1* contributes to yield improvement. RP is closely related to yield, quality, and tolerance to environmental stresses (Cheng et al., 2011). After flowering, soybeans advance to the pod-setting stage, and is the most vigorous period of soybean growth, which requires high amounts of water, nutrients, and light. During the seed-filling period, nutritional matter in the seeds gradually accumulates. An extremely short reproductive stage may lead to low levels of accumulation of dry matter in seeds, which in turn may severely impact production. However, when the reproductive stage is too long, the plants may be more vulnerable to the effects of cold snap at high latitudes, thereby leading to total loss of soybean yield. Therefore, in soybean breeding, we should balance the relation between RP and production. QTLs on chromosomes 6 and 11 may work together to regulate post-flowering biological processes. The previously reported QTL, photoperiod insensitivity 5–4, which is flanked by markers Satt244-BARC-041173-07927 and a physical position from 33,818,897 to 36,781,596, overlapped with *qR1-J* on chromosome 16 in our study (Liu et al., 2011). The interval of QTL *qR1-L* on chromosome 19 encompasses positions 44,953,211–45,862,765, where it coincides with the *Dt1* gene *Glyma.19G194300* (*TERMINAL FLOWER1, TFL1b*) (Liu et al., 2010; Tian et al., 2010). *TFL1* is a key regulator of flowering time and the development of the inflorescence meristem in *Arabidopsis thaliana* (Hanano and Goto, 2011). In this study, QTL *qR1-L* detected on chromosome 19 in the SE population may influence flowering time, and *qR1-L* may be controlled by the *Dt1* gene because the parent Suinong 14 possesses a dominant *Dt1* allele, whereas Enrei possesses a recessive *dt1* allele (Supplementary Figure 5). Functional characterization of how *Dt1* regulates flowering time and maturity, in addition to its role in controlling grow habit, will be very interesting and allow expansion of its applications in soybean yield improvement.

Fine mapping is a key step in target gene cloning, wherein recombinant individuals are screened using polymorphic markers within candidate regions and the genetic distance is shortened by correlation analysis between phenotypes and genotypes of the recombinant individuals. Searching for polymorphic markers based on parental resequencing data is particularly more important for fine mapping of genes near the centromeric region. Heterochromatic regions surrounding the centromeres suppresses recombination (Schmutz et al., 2010). Estimating the ratio of the genetic distance to the physical position in this region is generally difficult to obtain, and thus increasing the density of molecular markers in a region far from the centromere may be futile. To obtain multiple recombinant individuals, the fine mapping population should be large. A previous study showed that only 10 extremely precious recombinants were obtained from 13,760 F_2:5_ seeds during fine mapping of the *E1* gene near the centromeric region (Xia et al., 2012).

The accuracy of QTL mapping is also influenced by the genetic background and size of the study population, as well as the number of genetic markers employed in the analysis. In addition, investigating candidate genes based on the resequencing data of the parents is also influenced by sequencing depth and accuracy, as well as the accuracy of the reference genome assembly and annotation. The polymorphic loci information above helps in determining the genetic background of the two groups. The specific gene loci that actually result in phenotypic differences, their functional mechanisms, and the regulatory networks involved still need further investigations.

Fine mapping of the QTLs and validation of the potential candidate genes may be a reliable and feasible strategy for QTL cloning to isolate the candidate genes for the elucidation of the molecular mechanisms underlying photoperiod-regulated flowering and time to maturity. Map-based cloning of *qR1-C2, qR1-C1*, *qR1-J*, and *qRP-B1* and the functional characterization of these candidate genes are underway in our laboratory. In addition, QTL flanking markers are valuable tools for soybean molecular breeding to obtain cultivars that exhibit higher levels of adaptation and yield productivity.

## Author Contributions

FK designed the experiments. LK, SL, YW, CF, FW, HN, TS, SL, FZ, XL, XZ, and XY carried out the experiments. LK, BL, and FK analyzed the data. LK and FK wrote the paper.

## Conflict of Interest Statement

The authors declare that the research was conducted in the absence of any commercial or financial relationships that could be construed as a potential conflict of interest.

## Footnotes

1 https://phytozome.jgi.doe.gov/pz/portal.html#!info?alias=Org_Gmax

2 http://broadinstitute.github.io/picard

3 https://www.soybase.org/
