# Supplementary material for: Quantitative Trait Locus Mapping of Flowering Time and Maturity in Soybean Using Next-Generation Sequencing-Based Analysis
Source: Front Plant Sci. 2018 Jul 11;9:995. doi: 10.3389/fpls.2018.00995 (PMC6050445; doi:10.3389/fpls.2018.00995)
Supplement: Supplementary file 1 [file Data_Sheet_1.zip › Supplementary materials/Supplementary figures.pptx]

## Slide 1
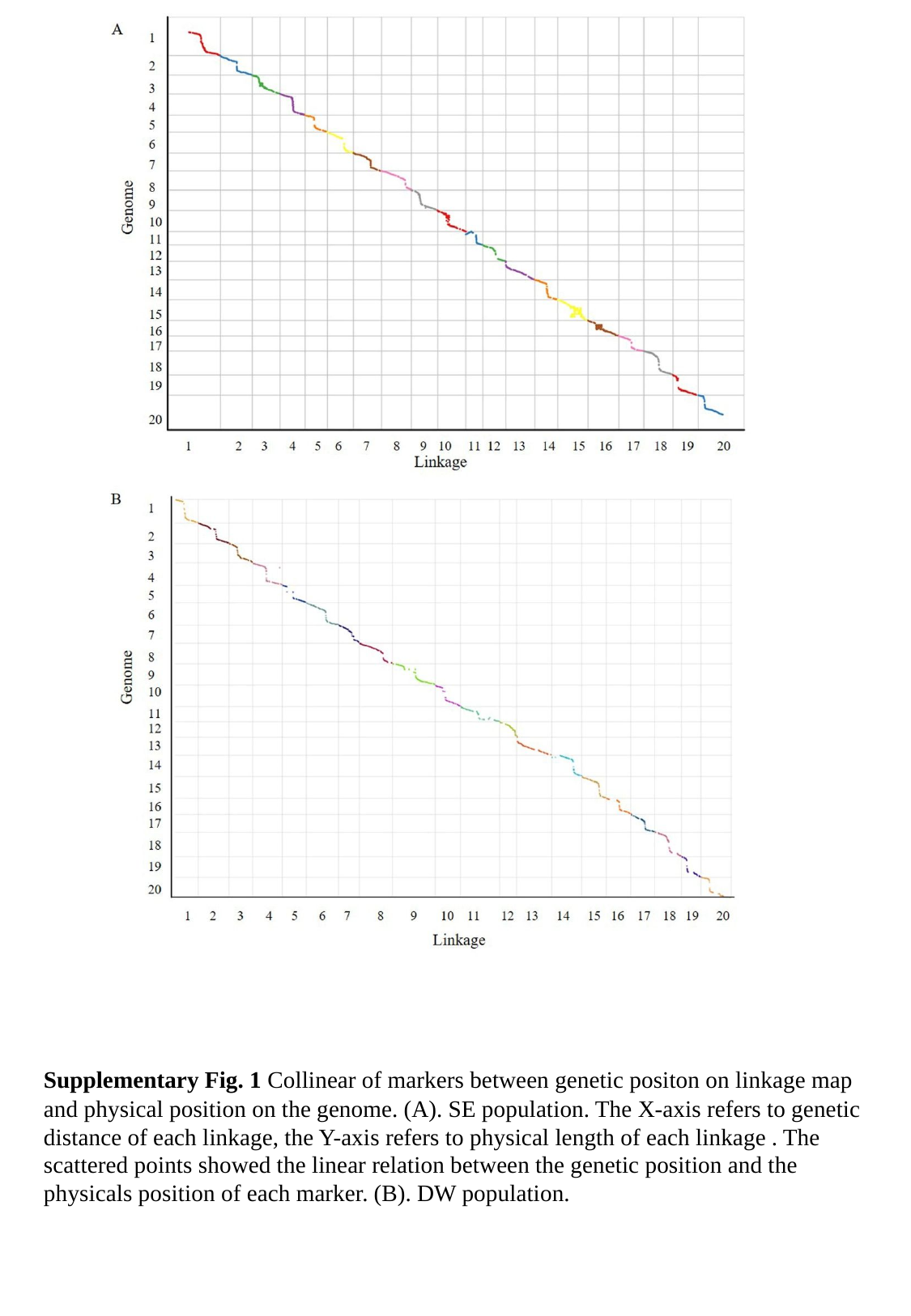

Supplementary Fig. 1 Collinear of markers between genetic positon on linkage map and physical position on the genome. (A). SE population. The X-axis refers to genetic distance of each linkage, the Y-axis refers to physical length of each linkage . The scattered points showed the linear relation between the genetic position and the physicals position of each marker. (B). DW population.

## Slide 2
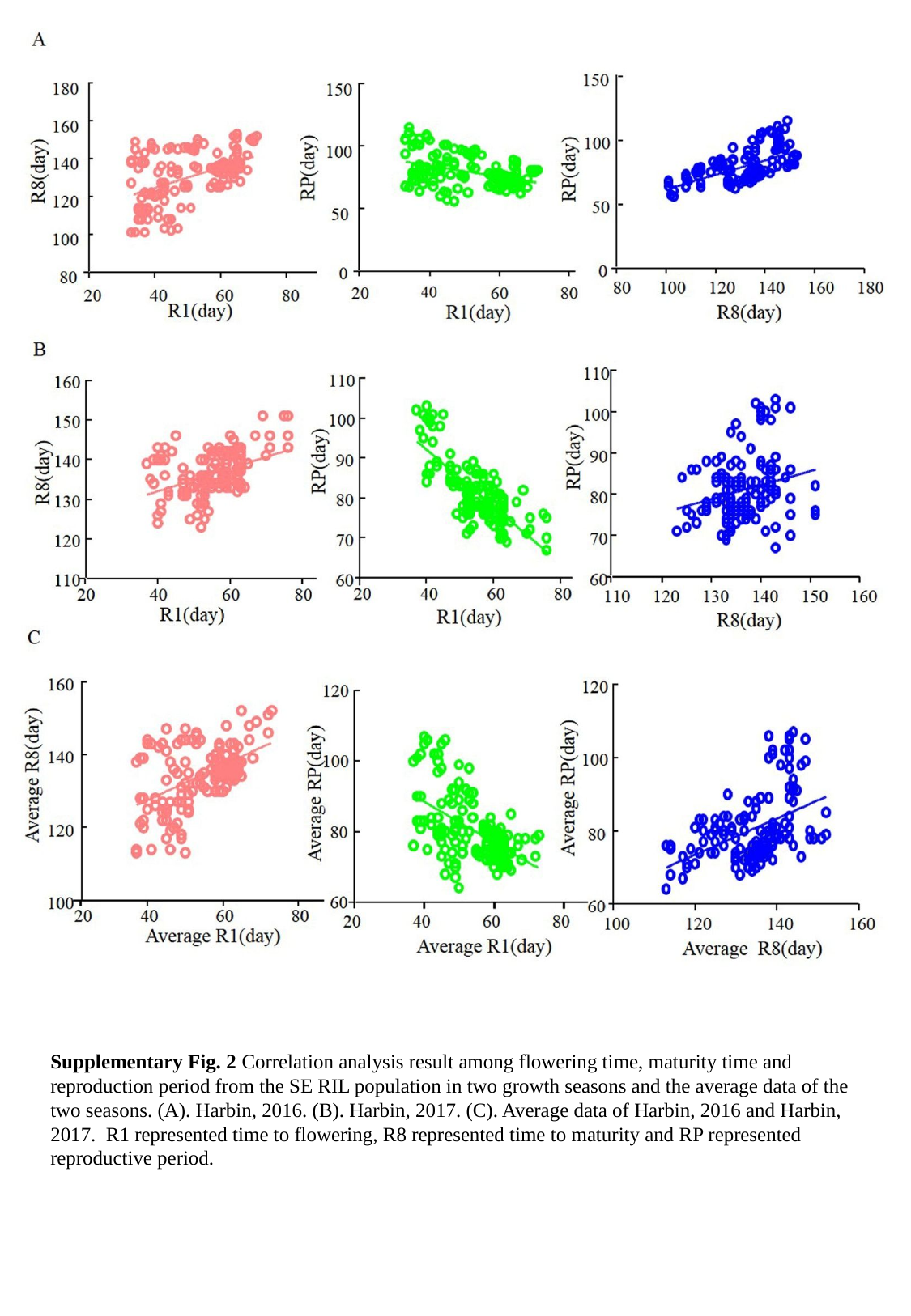

Supplementary Fig. 2 Correlation analysis result among flowering time, maturity time and reproduction period from the SE RIL population in two growth seasons and the average data of the two seasons. (A). Harbin, 2016. (B). Harbin, 2017. (C). Average data of Harbin, 2016 and Harbin, 2017. R1 represented time to flowering, R8 represented time to maturity and RP represented reproductive period.

## Slide 3
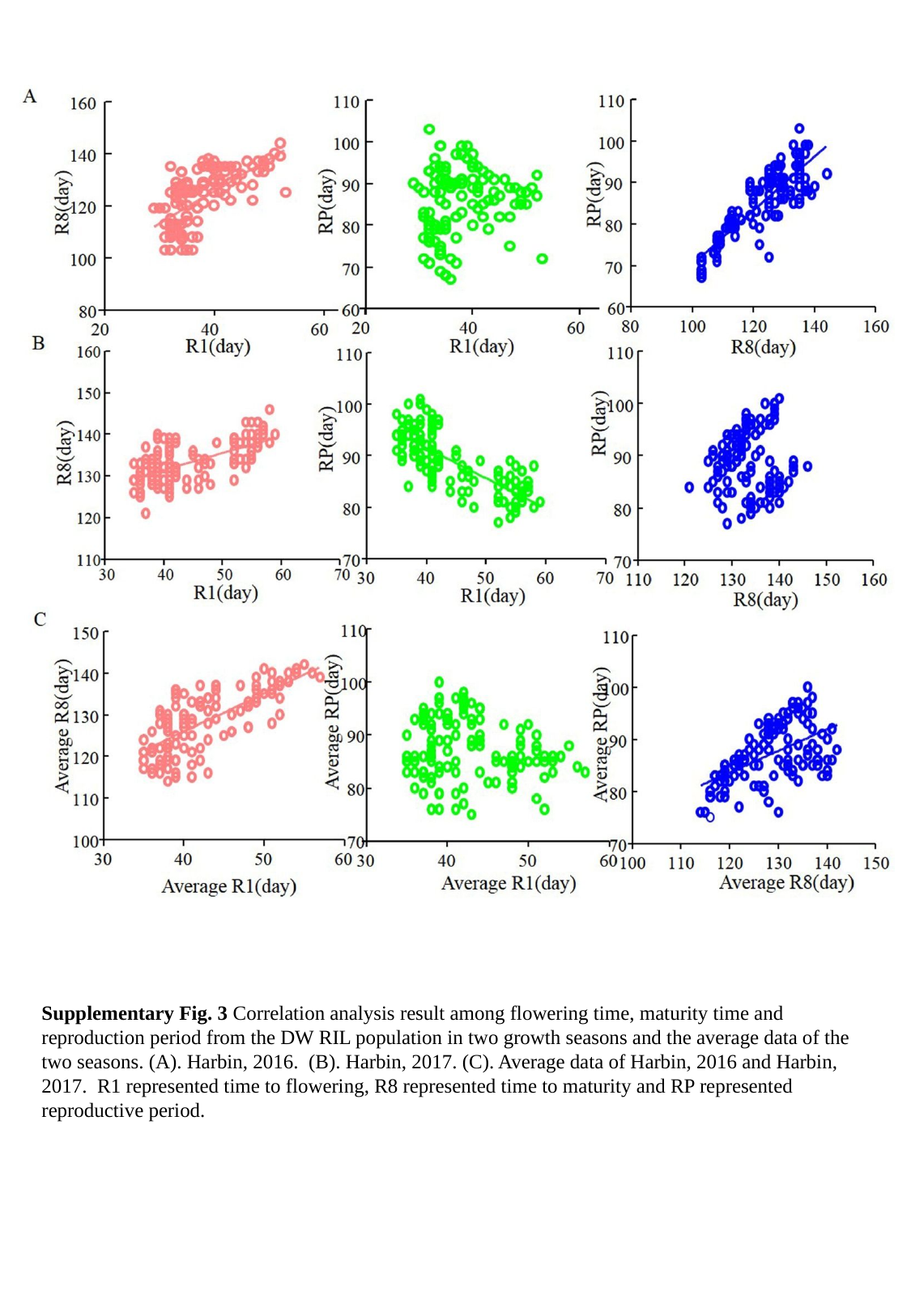

Supplementary Fig. 3 Correlation analysis result among flowering time, maturity time and reproduction period from the DW RIL population in two growth seasons and the average data of the two seasons. (A). Harbin, 2016. (B). Harbin, 2017. (C). Average data of Harbin, 2016 and Harbin, 2017. R1 represented time to flowering, R8 represented time to maturity and RP represented reproductive period.

## Slide 4
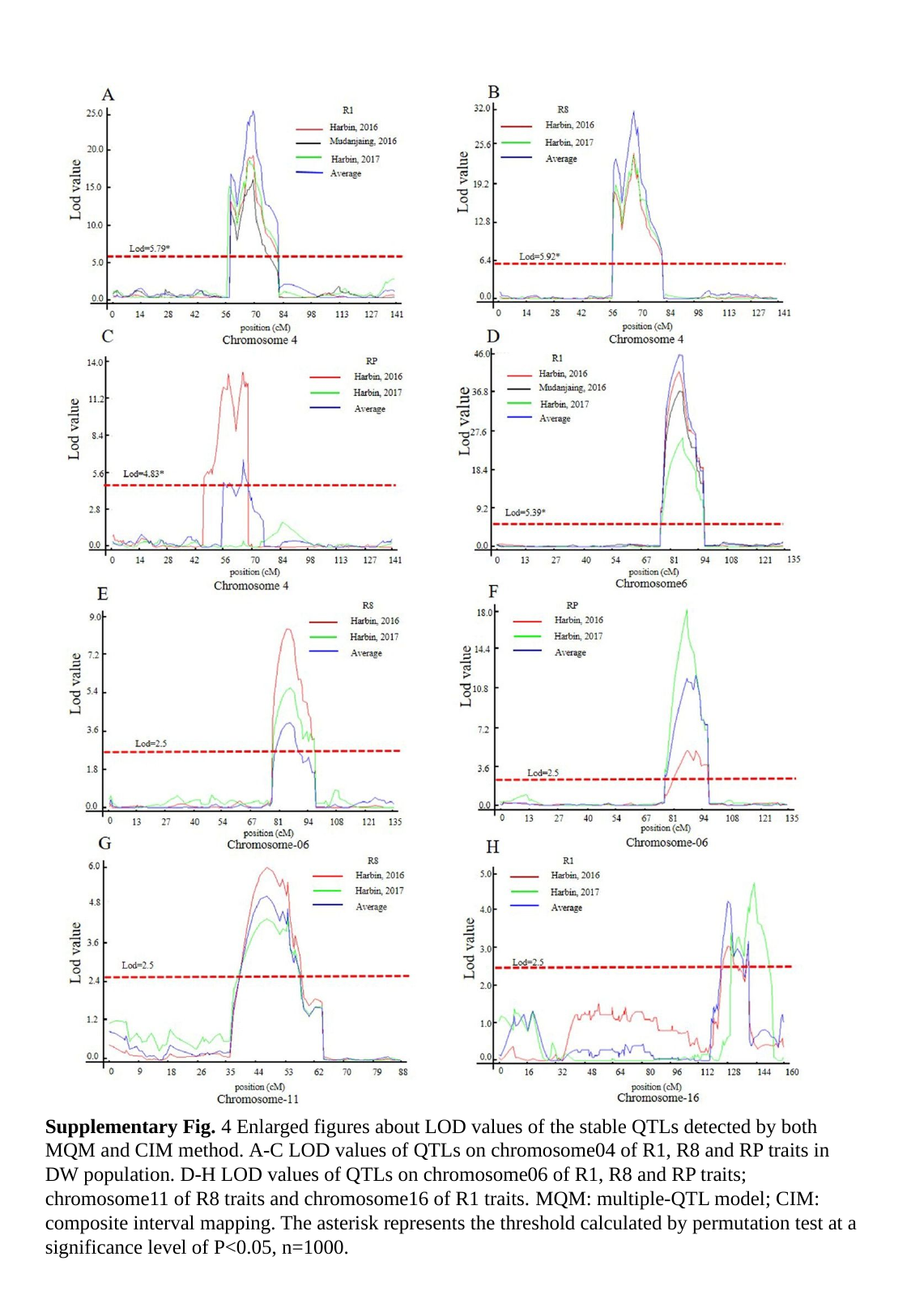

Supplementary Fig. 4 Enlarged figures about LOD values of the stable QTLs detected by both MQM and CIM method. A-C LOD values of QTLs on chromosome04 of R1, R8 and RP traits in DW population. D-H LOD values of QTLs on chromosome06 of R1, R8 and RP traits; chromosome11 of R8 traits and chromosome16 of R1 traits. MQM: multiple-QTL model; CIM: composite interval mapping. The asterisk represents the threshold calculated by permutation test at a significance level of P<0.05, n=1000.

## Slide 5
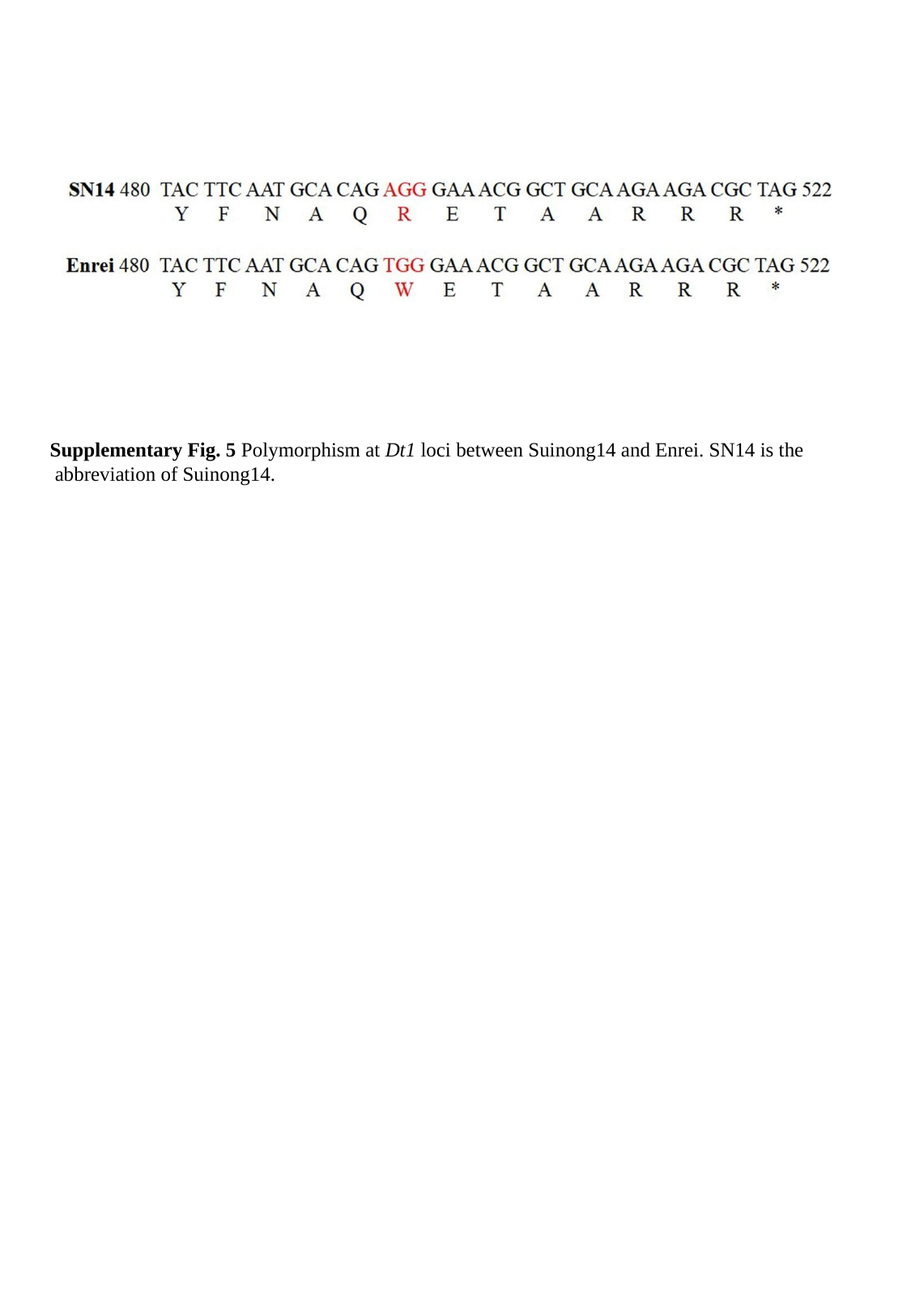

Supplementary Fig. 5 Polymorphism at Dt1 loci between Suinong14 and Enrei. SN14 is the
 abbreviation of Suinong14.
