# Supplementary material for: Quantitative Trait Locus Mapping of Flowering Time and Maturity in Soybean Using Next-Generation Sequencing-Based Analysis
Source: Front Plant Sci. 2018 Jul 11;9:995. doi: 10.3389/fpls.2018.00995 (PMC6050445; doi:10.3389/fpls.2018.00995)
Supplement: Supplementary file 1 [file Data_Sheet_1.zip › Supplementary materials/Supplementary material.docx]

***Supplementary Material***

**Quantitative trait locus (QTL) mapping of flowering time and maturity in soybean using next generation sequencing (NGS)-based analysis**

Lingping Kong^1,3,5^, Sijia Lu^2,5^, Yanping Wang^4,5^, Chao Fang^1,3,5^, Feifei Wang^1^, Haiyang Nan^2^, Tong Su^1,3^, Shichen Li^1,3^, Fengge Zhang^1,3^, Xiaoming Li^1^, Xiaohui Zhao^2^, Xiaohui Yuan^1^, Baohui Liu^1,2*^, Fanjiang Kong^1,2*^

^1^The Key Laboratory of Soybean Molecular Design Breeding, Northeast Institute of Geography and Agroecology, Chinese Academy of Sciences, Harbin, China

^2^School of Life Sciences, Guangzhou University, Guangzhou, China

^3^University of Chinese Academy of Sciences, Beijing, China

^4^Mudanjiang Branch of Heilongjiang Academy of Agricultural Sciences, Mudanjiang, China

^5^These authors contributed equally to this work.

*** Correspondence:**

Corresponding author

Fanjiang Kong, [kongfj@iga.ac.cn](mailto:kongfj@iga.ac.cn)

Baohui Liu, [liubh@neigaehrb.ac.c](mailto:liubh@neigaehrb.ac.c)n

**Supplementary Data**

**Supplementary Fig. 1** Collinear of markers between genetic positon on linkage map and physical position on the genome. (A). SE population. The X-axis refers to genetic distance of each linkage, the Y-axis refers to physical length of each linkage. The scattered points showed the linear relation between the genetic position and the physicals position of each marker. (B). DW population.

**Supplementary Fig. 2** Correlation analysis result among flowering time, maturity time and reproduction period from the SE RIL population in two growth seasons and the average data of the two seasons. (A). Harbin, 2016. (B). Harbin, 2017. (C). Average data of Harbin, 2016 and Harbin, 2017. R1 represented time to flowering, R8 represented time to maturity and RP represented reproductive period.

**Supplementary Fig. 3** Correlation analysis result among flowering time, maturity time and reproduction period from the DW RIL population in two growth seasons and the average data of the two seasons. (A). Harbin, 2016. (B). Harbin, 2017. (C). Average data of Harbin, 2016 and Harbin, 2017. R1 resented time to flowering, R8 represented time to maturity and RP represented reproductive period.

**Supplementary Fig. 4** Enlarged figures about LOD values of the stable QTLs detected by both MQM and CIM method. A-C LOD values of QTLs on chromosome04 of R1, R8 and RP traits in DW population. D-H LOD values of QTLs on chromosome06 of R1, R8 and RP traits; chromosome11 of R8 traits and chromosome16 of R1 traits. MQM (multiple-QTL model), CIM (composite interval mapping). The asterisk represents the threshold calculated by permutation test at a significance level of P<0.05, n=1000.

**Supplementary Fig. 5** Polymorphism at *Dt1* loci between Suinong14 and Enrei. SN14 is the abbreviation of Suinong14.

**Supplementary Table 1** Resequencing data analysis result of parental cultivars. Q30: Sequencing error of one read was 0.001, the corresponding quality value of the read was 30.

**Supplementary Table 2** Description of characteristics of the 20 linkage groups in the genetic map of SE RIL population. SE: abbreviation for the cross between Suinong 14 × Enrei. RIL: recombinant inbred line.

**Supplementary Table 3** Description of characteristics of the 20 linkage groups in the genetic map of DW RIL population. DW: abbreviation for the cross between Dongnong 50 × Williams 82.

**Supplementary Table 4** Correlation analysis result between phenotypes of two RIL populations. SE: abbreviation for the cross between Suinong 14 × Enre; DW: abbreviation for the cross between Dongnong 50 × Williams 82. Correlation analysis was carried out using the respective data of 2016 and 2017 in Harbin of the two RIL populations and the average data of the two years. R1: flowering time trait that means days from emergence to first open flower appeared on 50% of the plants in one line. R8: maturity time trait that means days from emergence to 95% of pods have turned their mature color on 50% of the plants in one line. RP: represents reproduction period and RP=R1-R8. Average R8: the average data of R8 in two environments (2016 and 2017 in Harbin). Average RP: the average data of RP in two environments (2016 and 2017 in Harbin).

**Supplementary Table 5** The threshold of LOD scores using 1000 times permutations test.

**Supplementary Table 6**Some reported commonality QTLs of growth period related traits.

**Supplementary Table 7** Other minor QTLs detected in SE population.

**Supplementary Table 8** Other minor QTLs detected in DW population.

**Supplementary Table 9** Candidate genes in the major QTL intervals of two RIL populations involving in photoperiod response progresses regulating flowering and reproduction.

**Supplementary Table 10** Polymorphic variations between parents within CDS in the major QTL interval of chromosome 4.

**Supplementary Table 11** Polymorphic variations between parents within gene in the major QTL interval of chromosome 6.

**Supplementary Table 12** Information of 25 candidate genes within QTL interval on chromosome 16. Annotation information of genes on chromosome 16 within the 1.5-LOD drop on either side of the peak marker in QTL interval according to Phytozome data base of Wm82.a2.v1. Only genes with amino acids polymorphism between parents were list in the table and detail information of polymorphism amino acids was not listed. Gene Start: start position of 5'UTR (Untranslated Regions) of one gene. Gene End: end position of 3'UTR of one gene.

**Supplementary Table 13** Genetic distance information of all markers used to construct genetic map of the SE population.

**Supplementary Table 14** Sequence and physical position information of all markers in SE population.

**Supplementary Table 15** Genetic distance and physical position information of all markers in DW population.

**Supplementary Table 16** Gene ontology (GO) analysis result of the candidate genes within the high confidential interval of the major QTL on chromosome 4.

**Supplementary Table 17** Gene ontology (GO) analysis result of the candidate genes within the high confidential interval of the major QTL on chromosome 6.
